# Raw and Cooked Vegetable Consumption and Risk of Cardiovascular Disease: A Study of 400,000 Adults in UK Biobank

**DOI:** 10.3389/fnut.2022.831470

**Published:** 2022-02-21

**Authors:** Qi Feng, Jean H. Kim, Wemimo Omiyale, Jelena Bešević, Megan Conroy, Margaret May, Zuyao Yang, Samuel Yeung-shan Wong, Kelvin Kam-fai Tsoi, Naomi Allen, Ben Lacey

**Affiliations:** ^1^Nuffield Department of Population Health (NDPH), University of Oxford, Oxford, United Kingdom; ^2^JC School of Public Health and Primary Care, The Chinese University of Hong Kong, Shatin, Hong Kong SAR, China; ^3^Population Health Sciences, University of Bristol, Bristol, United Kingdom; ^4^SH Big Data Decision Analytics Research Centre, The Chinese University of Hong Kong, Shatin, Hong Kong SAR, China

**Keywords:** vegetable intake, raw vegetable, cooked vegetable, cardiovascular diseases, UK biobank, cardiovascular mortality

## Abstract

**Objectives:**

Higher levels of vegetable consumption have been associated with a lower risk of cardiovascular disease (CVD), but the independent effect of raw and cooked vegetable consumption remains unclear.

**Methods:**

From the UK Biobank cohort, 399,586 participants without prior CVD were included in the analysis. Raw and cooked vegetable intakes were measured with a validated dietary questionnaire at baseline. Multivariable Cox regression was used to estimate the associations between vegetable intake and CVD incidence and mortality, adjusted for socioeconomic status, health status, and lifestyle factors. The potential effect of residual confounding was assessed by calculating the percentage reduction in the likelihood ratio (LR) statistics after adjustment for the confounders.

**Results:**

The mean age was 56 years and 55% were women. Mean intakes of raw and cooked vegetables were 2.3 and 2.8 tablespoons/day, respectively. During 12 years of follow-up, 18,052 major CVD events and 4,406 CVD deaths occurred. Raw vegetable intake was inversely associated with both CVD incidence (adjusted hazard ratio (HR) [95% CI] for the highest vs. lowest intake: 0.89 [0.83–0.95]) and CVD mortality (0.85 [0.74–0.97]), while cooked vegetable intake was not (1.00 [0.91–1.09] and 0.96 [0.80–1.13], respectively). Adjustment for potential confounders reduced the LR statistics for the associations of raw vegetables with CVD incidence and mortality by 82 and 87%, respectively.

**Conclusions:**

Higher intakes of raw, but not cooked, vegetables were associated with lower CVD risk. Residual confounding is likely to account for much, if not all, of the observed associations. This study suggests the need to reappraise the evidence on the burden of CVD disease attributable to low vegetable intake in the high-income populations.

## Introduction

There exists a large body of research evidence to suggest that a high vegetable intake may protect against a wide range of health outcomes, including cardiovascular disease (CVD) (1, 2). Although dietary guidelines have consistently recommended a high consumption of vegetables to the general population (3, 4) as a source of beneficial macronutrients and micronutrients, such as dietary fiber, vitamins, and phytochemicals (5), it is estimated that inadequate vegetable consumption accounts for about 1.5 million premature deaths from CVD alone each year (6).

However, little is known about the independent effects of cooked vegetables and raw vegetables on health outcomes. Previous epidemiological studies have demonstrated inconsistent findings. The EPIC study (7) of 450,000 participants recruited across Europe found that both cooked and raw vegetable intake was associated with lower CVD mortality and all-cause mortality. The PURE study (8) of 135,000 participants reported an inverse association with all-cause mortality for raw vegetable intake, but not for cooked vegetable intake, and neither cooked nor raw vegetable intake was associated with CVD incidence. An Australian cohort study (9) of 150,000 participants reported that only cooked vegetable intake was associated with lower overall mortality, but did not investigate cardiovascular outcomes. The reason for the discrepancies in these findings is unclear, but may reflect variation in the dietary patterns between populations and also methodological differences, such as dietary assessment methods and insufficient adjustment for potential confounders.

The UK Biobank is a cohort of half-million participants with over a decade of follow-up (10). A wide range of participant characteristics were measured at baseline using standardized methods, minimizing measurement error and allowing for adjustment for a broad set of potential confounders. During follow-up, a large number of incident CVD and CVD deaths have been recorded, allowing for well-powered epidemiological investigations on cardiovascular outcomes (11). The objective of this study was to examine the effect of vegetable intake, and specifically the independent effects of raw and cooked vegetable intake, on CVD incidence and mortality in UK Biobank.

## Methods

### Study Design and Participants

The UK Biobank is a population-based prospective cohort study (10). Between 2006 and 2010, half-million participants aged 40–69 years were recruited across England, Wales, and Scotland. Participants attended assessment centers, during which time they completed a touchscreen questionnaire that collected information on sociodemographic characteristics, lifestyle, health status, medication use, reproductive history, and environmental factors. In addition, anthropometric and other physical measures were taken using standardized procedures, and blood, urine, and saliva samples were collected.

The health of the participants was followed-up *via* linkage to hospitalization databases (the National Health Service [NHS] Hospital Episode Statistics for participants in England; the Scottish Morbidity Record for participants in Scotland; and the Patient Episode Database for participants in Wales) and national death registries (NHS Information Center for participants in England and Wales; and NHS Central Registry for participants in Scotland). UK Biobank was approved by the North West Multicenter Research Ethics Committee, the National Information Governance Board for Health and Social Care in England and Wales, and the Community Health Index Advisory Group in Scotland. All the participants provided the informed consent.

**Figure 1:**
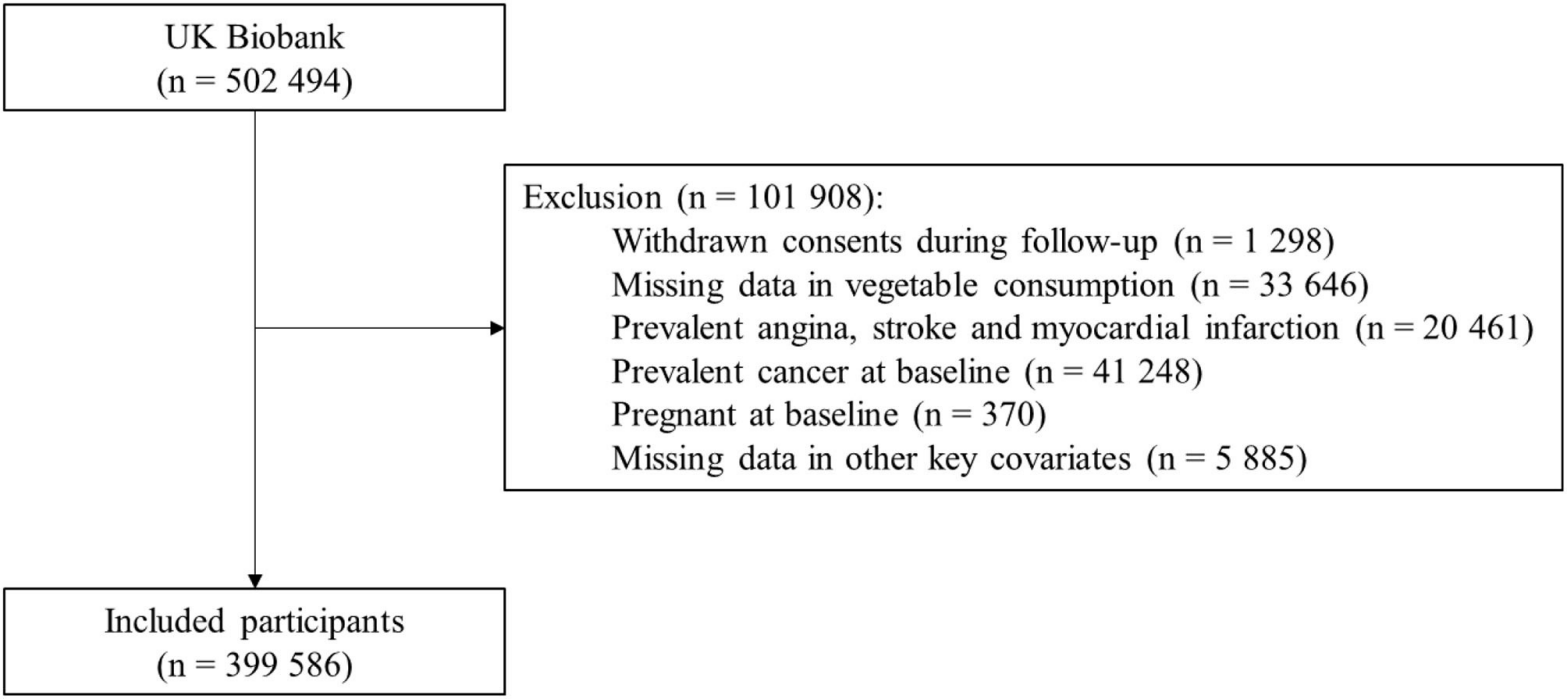
Flowchart of participant included in the main analysis.

This study excluded participants that withdrew their consents during follow-up, had missing data on vegetable intake, had prior CVDs, had conditions likely to change dietary patterns (e.g., pregnancy and cancer). Furthermore, 5,885 participants had missing data on other key covariates (body mass index [BMI], meat consumption, and Townsend deprivation index), and were excluded. In total, 399,586 participants were included in the analysis (Figure 1).

### Measurement of Exposures and Outcomes

Information was collected at baseline on the total daily intake of raw vegetables and cooked vegetables. Participants were asked in the dietary questionnaire “*On average how many heaped tablespoons of salad or raw vegetables would you eat per day? (including lettuce, tomato in sandwiches)*” and “*On average how many heaped tablespoons of cooked vegetables would you eat per day? (do not include potatoes)*”. Total vegetable intake was calculated as the sum of raw and cooked vegetable intakes. Vegetable intake was categorized into four levels, using cutoff values of 0, 1–2, 3–4, and ≥ 5 tablespoons/day for raw and cooked vegetable intake, and cutoff values of 0–1, 2–3, 4–7, and ≥ 8 for total vegetable intake. Previous analyses have demonstrated high repeatability and validity of vegetable consumption measured in this baseline dietary questionnaire: repeatability over a 4-year period is 82% for cooked vegetables and 72% for raw vegetables, with high agreement also observed when compared with 24-h dietary assessment (12).

The primary outcomes were CVD incidence and mortality. The secondary outcomes were incident myocardial infarction (MI), incident stroke, and all-cause mortality. Incident CVD was defined as hospitalization or death from MI or stroke (13). CVD mortality was defined as death due to any CVD. For analyses of disease incidence, participants were censored at the date of hospitalization, date of death, or last date of follow-up (March 31, 2021 for participants from England and Scotland, and February 28, 2018 for participants from Wales), whichever occurred first. In the mortality analysis, participants were censored at the date of death or last date of follow-up (February 28, 2021), whichever occurred first. Health outcomes were defined using the International Classification of Disease (ICD) codes. The exact ICD codes used are shown in Supplementary Table 1.

### Statistical Analysis

Cox proportional hazard models were used to yield hazard ratios (HR) and 95% CI for the associations between health outcomes and vegetable intake. Models were adjusted by age (<50, 50–60, ≥ 60 years), sex, ethnicity, and region, and adjusted for educational attainment, Townsend deprivation index (continuous), hypertension, diabetes, physical activity level, smoking, alcohol consumption, BMI (continuous), use of mineral supplements, use of vitamin supplements, aspirin/ibuprofen, antihypertensive drugs, statins, insulin treatment, intake of fresh fruits, red meat, processed meat, oily fish, and non-oily fish. The definition and measurement of the covariates are shown in the Supplementary Materials. The lowest intake level was used as the referent group. Test of the linear trend was obtained by fitting the mean values of each vegetable intake level. The proportional hazards assumption was assessed using scaled Schoenfeld residuals (no violation was found in this study). Raw and cooked vegetable intake were mutually adjusted when investigating their independent effects. Variance inflation factor values were used to examine potential multicollinearity.

We calculated the increase in the likelihood ratio (LR) chi-squared statistics on the addition of the vegetable intake term (raw, cooked, and total) to the Cox models with various levels of adjustment of potential confounders. This provides a quantitative measure of the extent to which vegetable intake improves risk prediction for the outcome in different models. Comparisons of the changes in the LR chi-squared statistic between a model with minimal adjustments (e.g., age, sex, ethnicity, and region) to those with a more comprehensive set of confounders (“fully-adjusted” models) is therefore measure of the extent to which the confounders account for minimally adjusted associations between vegetable intake and the outcome of interest. Furthermore, given that many confounders are measured imperfectly, the proportional change in this LR chi-squared statistic is a semiquantitative method of assessing for residual confounding, as models with perfectly measured confounders would be expected to further reduce the LR chi-squared statistic in fully adjusted models (14). More details are shown in the Supplementary Materials.

For sensitivity analysis, we first excluded participants who developed the outcomes of interest during the first 2 years of follow-up, to minimize reverse causation. Second, we investigated the effect of the proportion of raw vegetables in total vegetable intake (raw vegetables divided by total vegetables), conditional on total vegetable intake and other covariates, after excluding the participants with the total vegetable intake of *zero* tablespoon/day (*n* = 5,304). We conducted subgroup analysis based on ethnicity (White vs. non-White), to examine potential ethnic differences in the associations. All the analysis were performed using R (version 3.6.0; R Development Core Team, Vienna, Austria).

## Results

After exclusion, 399,586 participants were included in the main analysis (Figure 1). The baseline characteristics of these participants are shown in Table 1 (Supplementary Table 2). The mean age of participants was 56.1 (SD 8.1) years, 55.4% were women, and 90.9% were of White ethnicity. Mean BMI was 27.3 (4.7) kg/m^2^, 41.3% reported high levels of physical activity, and 4.7% had a self-reported history of diabetes. Mean intakes of total vegetables, raw vegetables, and cooked vegetables were 5.0 (3.4), 2.3 (2.2), and 2.8 (2.2) tablespoons/day, respectively; the distributions of total, raw and cooked vegetable intakes are shown in Supplementary Figure 1.

**Table 1:** Baseline characteristics of the 399,586 participants in the main analysis, by total vegetable consumption.

	**≤1 tablespoon/day (*n* = 15 902)**	**2–3 tablespoons/day (*n* = 109 536)**	**4–7 tablespoons/day (*n* = 216 499)**	**≥8 tablespoons/day (*n* = 57 649)**	**Overall (*n* = 399 586)**
Female (%)	6 174 (38.8)	54 948 (50.2)	126 375 (58.4)	33 997 (59.0)	221 494 (55.4)
Age (years)	54.0 (8.1)	55.3 (8.2)	56.5 (8.0)	56.4 (8.0)	56.1 (8.1)
Total vegetable intake (tablespoons/day)	0.7 (0.5)	2.6 (0.5)	5.1 (1.0)	10.7 (5.0)	5.0 (3.4)
Raw vegetable intake (tablespoons/day)	0.1 (0.3)	0.9 (0.6)	2.2 (1.1)	5.5 (3.5)	2.3 (2.5)
Cooked vegetable intake (tablespoons/day)	0.5 (0.5)	1.7 (0.6)	2.8 (1.0)	5.3 (3.4)	2.8 (1.9)
White ethnicity (%)	14 782 (93.3)	104 731 (95.9)	206 372 (95.6)	52 143 (90.9)	378 028 (94.9)
Townsend Deprivation index^*^	−0.2 (3.5)	−1.4 (3.0)	−1.5 (2.9)	−1.1 (3.1)	−1.4 (3.0)
University educated (%)	3,321 (21.3)	37,040 (34.3)	73,733 (34.6)	19,483 (34.5)	133,577 (34.0)
Body mass index (kg/m^2^)	28.0 (5.2)	27.2 (4.7)	27.3 (4.7)	27.4 (4.8)	27.3 (4.7)
Current smoker (%)	3 485 (22.0)	11 828 (10.8)	19 427 (9.0)	5 506 (9.6)	40 246 (10.1)
Current drinker (%)	13 817 (87.1)	101 873 (93.1)	201 964 (93.3)	52 123 (90.5)	369 777 (92.6)
High physical activity level (%)^†^	3 971 (32.2)	31 459 (35.0)	75 528 (42.5)	24 328 (51.0)	135 286 (41.3)
Self-reported hypertension (%)	4 172 (26.2)	26 482 (24.2)	55 071 (25.4)	15 131 (26.2)	100 856 (25.2)
Self-reported diabetes (%)	994 (6.2)	4 859 (4.4)	9 904 (4.6)	3 009 (5.2)	18 766 (4.7)
Regular use of aspirin/ibuprofen (%)	4 065 (25.6)	26 039 (23.8)	53 667 (24.8)	14 394 (25.0)	98 165 (24.6)
Regular use of mineral supplement (%)	2 869 (18.0)	25 789 (23.5)	61 980 (28.6)	17 955 (31.1)	108 593 (27.2)
Regular use of vitamin supplement (%)	1 760 (11.1)	13 223 (12.1)	30 534 (14.1)	9 756 (16.9)	55 273 (13.8)
Use of antihypertensive drugs (%)	938 (5.9)	8 133 (7.4)	20 449 (9.4)	5 628 (9.8)	35 148 (8.8)
Use of statin (%)	696 (4.4)	5 426 (4.9)	13 443 (6.2)	3 822 (6.6)	23 387 (5.9)
Use of insulin (%)	58 (0.4)	375 (0.3)	855 (0.4)	275 (0.5)	1 563 (0.4)
Fruit intake ≥5 pieces/day (%)	684 (4.3)	4 659 (4.3)	15 781 (7.3)	10 076 (17.5)	31 200 (7.8)
Oily fish intake >1 times/week (%)	3 267 (20.7)	39 335 (36.0)	88 514 (41.0)	21 682 (37.7)	72 515 (18.2)
Non-oily fish intake >1 times /week (%)	1 565 (9.9)	13 441 (12.3)	37 587 (17.4)	13 698 (23.8)	66 291 (16.6)
Processed meat intake ≥2 times/week (%)	6 949 (43.8)	38 331 (35.0)	62 278 (28.8)	14 132 (24.5)	121 690 (30.5)
Red meat intake (times/week)	2.0 (1.6)	2.1 (1.4)	2.1 (1.4)	2.0 (1.6)	2.1 (1.4)

*Data are mean (SD) or frequency (percentage). Vegetable consumption was self-reported in number of heaped tablespoons/day. People with baseline angina, stroke, myocardial infarction, cancer, pregnancy, and missing data on vegetable consumption and other important covariates were excluded*.

* *Area-level measure of material deprivation*.

† *High physical activity defined based on International Physical Activity Questionnaire and WHO guideline*.

Participants with higher levels of total vegetable intake were more likely to be women, better educated, and residents of an affluent area, with lower mean BMI and higher levels of physical activity, and less likely to be smokers. Raw and cooked vegetable intake were weakly correlated (Pearson correlation coefficient = 0.30). Variance inflation factor values for raw and cooked vegetable intake were 1.32 and 1.29, respectively, indicating very low collinearity (< 10). Supplementary Tables 3, 4 showed the baseline characteristics of the participants across different raw vegetable intake levels and the cooked vegetable intake levels, respectively. The distributions of baseline characteristics by raw and cooked vegetable intake were similar to the distributions by total vegetable intake.

**Figure 2:**
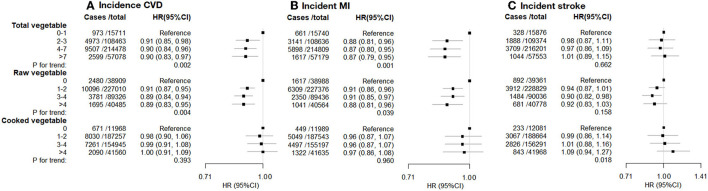
**(A)** incident CVD. **(B)** incident MI. **(C)** Incident stroke. Incident cardiovascular disease (CVD), myocardial infarction (MI), and stroke vs. vegetable consumption. Hazard ratios (HR; fully adjusted models) and 95% confidence interval (CI) by the level of total, raw, and cooked vegetable consumption (heaped tablespoons/day), relative to the lowest (reference) consumption level. Exclusions as in Table 1. The model was stratified by age (<50, 50–9660, 60 years), sex, ethnicity, and region, and adjusted for educational attainment, Townsend deprivation index (continuous), hypertension, diabetes, physical activity level, smoking, alcohol consumption, BMI (continuous), use of mineral supplements, use of vitamin supplements, aspirin/ibuprofen, antihypertensive drugs, statins, insulin treatment, intake of fresh fruits, red meat, processed meat, oily fish and non-oily fish.

During a median follow-up of 12.1 years for CVD incidence outcomes, 18,052 participants developed CVD (11,317 MI and 6,969 strokes). There was an inverse association between incident CVD and total and raw vegetable intake, but not cooked vegetable intake (Figure 2; Supplementary Figure 2). Compared with the lowest level of total vegetable intake, the highest level was associated with 10% lower CVD incidence (HR [95% CI] 0.90 [0.83–0.97]). Higher intake of raw vegetable intake was inversely associated with incident CVD (HR [95% CI] for the highest vs. lowest intake: 0.89 [0.83–0.95]) and incident MI (0.88 [0.81–0.96]; Figure 2), whereas cooked vegetable intake showed null associations with incident CVD (1.00 [0.91–1.09]) or incidence MI (0.97 [0.86–1.08]). We noted a potential inverse association between raw vegetable intake and incident stroke, although this was not statistically significant. No evidence was found for an association between incident stroke and total or cooked vegetable intakes (Figure 2).

**Figure 3:**
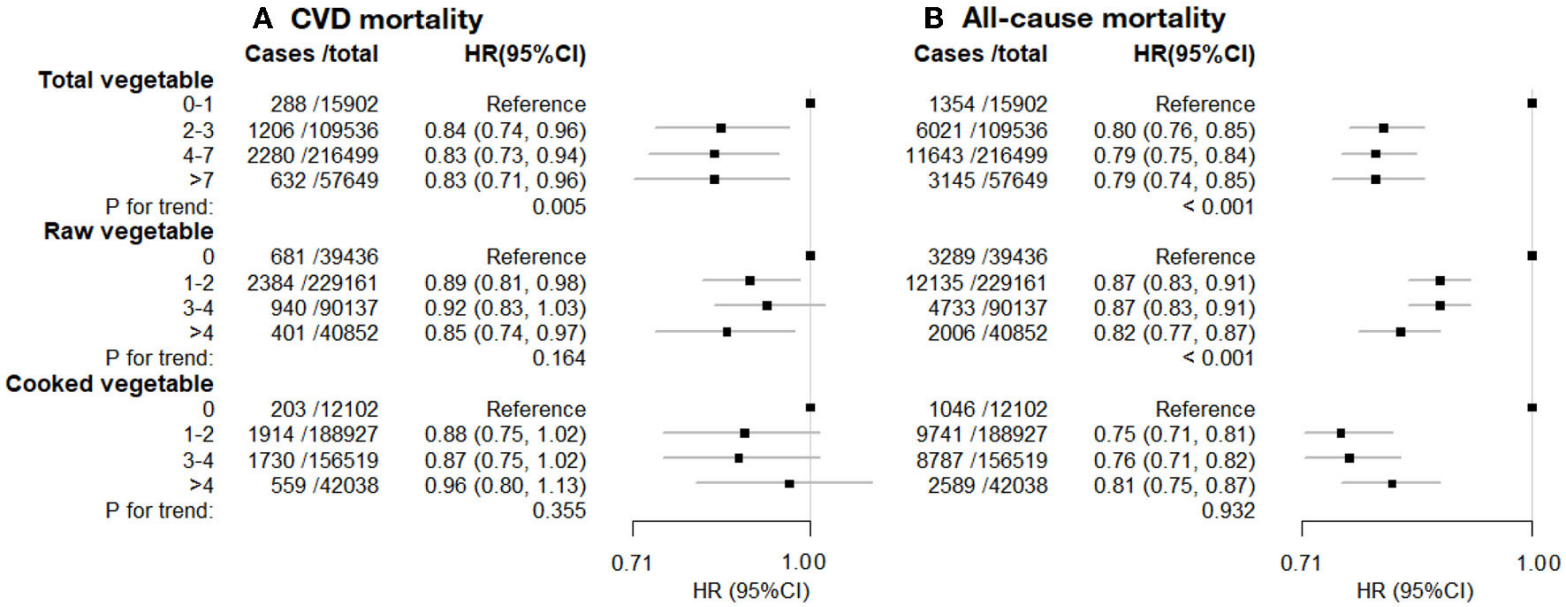
**(A)** CVD mortality. **(B)** All-cause mortality. Cardiovascular disease (CVD) mortality and all-cause mortality vs. vegetable consumption. Hazard ratios (HR; fully adjusted models) and 95% confidence interval (CI) by the level of total, raw, and cooked vegetable consumption (heaped tablespoons/day), relative to the lowest (reference) consumption level. Exclusions as in Table 1. The model was stratified by age (<50, 50–60, 60 years), sex, ethnicity, and region, and adjusted for educational attainment, Townsend deprivation index (continuous), hypertension, diabetes, physical activity level, smoking, alcohol consumption, BMI (continuous), use of mineral supplements, use of vitamin supplements, aspirin/ibuprofen, antihypertensive drugs, statins, insulin treatment, intake of fresh fruits, red meat, processed meat, oily fish and non-oily fish.

During a median follow-up of 12.0 years for mortality outcomes, 13,398 participants died, of which 2,589 deaths were due to CVD. Consuming 2 or more heaped tablespoons/day of total vegetables was associated with a lower risk of CVD mortality (HR [95%CI] for the highest vs. lowest intake: 0.83 [0.71–0.96]), but there was little evidence of a trend in risk with higher levels of intake (Figure 3). Similarly, there was evidence of an inverse association of CVD mortality with raw vegetable intake (0.85 [0.74–0.97]) but little evidence of a trend (*p* = 0.164), and there was no evidence of an association of CVD mortality with cooked vegetables. For all-cause mortality, there was a strong inverse association with eating some vegetables (1 or more tablespoons of raw or cooked vegetables per day), and strong evidence of a trend with increasing raw vegetable intake (*p* < 0.001) but not cooked vegetables (*p* = 0.932).

Progressive adjustment for potential confounders attenuated HR estimates and substantially reduced the LR chi-squared statistics in adjusted models (Table 2). For models of CVD incidence and raw vegetable intake, covariate adjustment attenuated HR (highest vs. lowest intake groups) from 0.79 (0.74–0.84) to 0.88 (0.83, 0.94), with the LR chi-squared statistic declining by 81.9%. This substantial attenuation suggests that were the potential confounders measured perfectly, much, if not all, of the observed association with reported vegetable intake, would be explained by residual confounding, although one cannot rule out the possibility of a true causal protective effect. Similar findings were observed for MI, CVD mortality, and all-cause mortality with both raw and cooked vegetable intake, with the proportional changes in the LR chi-squared statistic of about 80% or more (Table 2, Supplementary Table 5). Adjustment for socioeconomic (including educational attainment, and Townsend deprivation index) and lifestyle factors (including physical activity, smoking, drinking, use of mineral supplements, use of vitamin supplements, fruit intake, oily fish intake, non-oily fish intake, red meat intake, and processed meat intake) results in most of the reductions in LR chi-squared statistic, while further adjustment for BMI and baseline health status resulted in only slight further reductions (Supplementary Table 5), suggesting that the observed associations are likely to be accounted for by residual confounding from socioeconomic status and lifestyle factors.

**Table 2:** Associations between vegetable intake with CVD incidence, myocardial infarction incidence, stroke incidence, CVD mortality and all-cause mortality in basic model and fully-adjusted model.

	**Basic model**		**Fully-adjusted model**		**Attenuation (% reduction in χ^2^)^†^**
	**Improvement in fit (χ^2^)**	HR (95% CI)^*^	**Improvement in fit (χ^2^)**	HR (95% CI)^*^	
**CVD incidence**					
Total vegetable intake	87.8	0.74 (0.69, 0.80)	10.1	0.90 (0.83, 0.97)	88.6
Raw vegetable intake	127.9	0.79 (0.74, 0.84)	23.2	0.89 (0.83, 0.95)	81.9
Cooked vegetable intake	53.0	0.77 (0.71, 0.84)	1.5	1.00 (0.91, 1.09)	97.2
**MI incidence**					
Total vegetable intake	75.1	0.71 (0.65, 0.78)	11.1	0.87 (0.79, 0.95)	85.2
Raw vegetable intake	88.8	0.78 (0.72, 0.84)	13.3	0.88 (0.81, 0.96)	85.0
Cooked vegetable intake	42.8	0.74 (0.67, 0.83)	0.6	0.97 (0.86, 1.08)	98.5
**Stroke incidence**					
Total vegetable intake	18.8	0.84 (0.74, 0.95)	2.2	1.01 (0.89, 1.15)	88.1
Raw vegetable intake	31.7	0.85 (0.77, 0.94)	5.6	0.92 (0.83, 1.03)	82.2
Cooked vegetable intake	19.1	0.87 (0.75, 1.01)	6.1	1.09 (0.94, 1.27)	68.3
**All CVD mortality**					
Total vegetable intake	58.2	0.63 (0.55, 0.73)	8.0	0.83 (0.71, 0.96)	86.3
Raw vegetable intake	63.8	0.74 (0.65, 0.84)	8.2	0.85 (0.74, 0.97)	87.2
Cooked vegetable intake	53.9	0.67 (0.57, 0.78)	6.3	0.96 (0.80, 1.13)	88.4
**All-cause mortality**					
Total vegetable intake	298.7	0.61 (0.57, 0.65)	57.9	0.80 (0.74, 0.85)	80.6
Raw vegetable intake	352.7	0.69 (0.65, 0.73)	57.3	0.82 (0.77, 0.87)	83.8
Cooked vegetable intake	347.8	0.57 (0.53, 0.61)	72.0	0.81 (0.75, 0.87)	79.3

*Improvement in the prediction of relative risk (LR chi-squared statistic) by the addition of the given vegetable intake term to the basic model (in which the relative risk depends only on age, sex, ethnicity, and region), and to the fully-adjusted models with all major potential confounders, including mutual adjustment for raw and cooked vegetables, as described in the Methods*.

* *Hazard ratio for the highest vs. lowest vegetable intake group*.

† *Proportional reduction in chi-squared for the improvement in model fit relative to the basic model, equivalent to the proportion of the association attenuated by the potential confounders*.

In the sensitivity analyses, when adjusting for total vegetable intake, a higher proportion of raw vegetable intake in total vegetable intake was associated with lower CVD incidence and all-cause mortality, but not with other outcomes (Supplementary Table 6). Furthermore, excluding the participants who had outcome events within the first 2 years of follow-up did not materially change the main findings (Supplementary Table 7). Subgroup analyses restricted to White participants (*n* = 378,028) showed similar results to the primary analysis (Supplementary Table 8); and there was no evidence that the associations differed from those of non-White ethnicity, although there were substantially fewer non-White participants (*n* = 21,558) (Supplementary Table 9), and as such limited power to assess for heterogeneity.

## Discussion

In this large prospective cohort study, total vegetable intake was associated with reduced risks of CVD incidence, CVD mortality, and all-cause mortality. When assessing the independent effect of raw and cooked vegetable intake, only raw vegetable intake showed inverse associations with CVD outcomes, whereas cooked vegetables showed no association. However, given the large reductions in the predictive values of total and raw vegetable intake after adjustment for socioeconomic and lifestyle factors, residual confounding is likely to account for much, if not all, of the remaining associations.

The modest inverse associations of total vegetable intake with CVD outcomes and all-cause mortality in our analyses are consistent with previous large-scale observational evidence. For example, a meta-analysis of 24 cohort studies estimated that high vegetable intake reduced all-cause mortality by about 13% (relative risk 0.87 [0.82–0.92]) (15). Previous systematic reviews showed total vegetable consumption was associated with a risk reduction in CVD incidence by 11 (15) to 18% (16), similar to the ~10% lower risk in this study. Our findings of the inverse association with MI are also in line with published meta-analyses with effect sizes ranging from 9 to 15% (15–17). Although previous studies have also demonstrated an association with a reduced risk of stroke (15–17), we did not find sufficient evidence for such an association.

In contrast to a large number of studies on total vegetable intake, there is limited evidence on the independent effect of raw and cooked vegetables on all-cause mortality. Aune et al. (15) conducted a meta-analysis that found cooked vegetable was associated with 13% (relative risk 0.87 [0.80–0.94]) lower risk of all-cause mortality, and raw vegetable was associated with 12% (relative risk 0.88 [0.79–0.98]) lower risk of mortality, although the analyses of raw and cooked vegetables were not mutually adjusted. Studies that have attempted to assess the independent effects of raw and cooked vegetable intakes on all-cause mortality have reported conflicting results. Our results are broadly consistent with the EPIC study (7), in which both raw vegetable intake and cooked vegetable intake were associated with reduced risk of all-cause mortality. By contrast, the PURE study (8) reported an inverse association with all-cause mortality for raw vegetable intake, but not for cooked vegetable intake, while an Australian cohort study (9) reported that only cooked vegetable intake was associated with lower overall mortality. The characteristics and main findings of these studies are summarized in Supplementary Table 10.

In this study, cooked vegetable intake and raw vegetable intake showed different associations with cardiovascular outcomes. We found inverse associations of raw vegetables with CVD incidence and mortality, but null associations with cooked vegetables. This is consistent with the MORGEN study, a Dutch cohort (18), in which raw, but not processed, vegetables were associated with a reduced risk of ischemic stroke. In the EPIC cohort (7), there was a stronger inverse association of CVD mortality with raw than cooked vegetables. Whereas the PURE study (8) found no evidence of an association of CVD and raw vegetable intake, and high intakes levels of cooked vegetable was positively associated with CVD incidence.

Previous studies that reported associations of higher levels of vegetable intake with lower risk of CVD have proposed various mechanisms by which these associations might be mediated. For example, it has been suggested that diets high in vegetables have, on average, fewer calories and replace foods that are high in fat, sodium, and glycemic load (15, 19). It has also been hypothesized that the lower risk might be mediated by micronutrients, namely, higher intake of vitamins, polyphenols, and antioxidant compounds (2, 5), which are required for regulating various biological processes, including anti-oxidation, anti-inflammation, lipid metabolism, and endothelial function (20). As for the different associations of raw and cooked vegetables observed in this and other studies, several possible mechanisms have been proposed in previous studies. First, it has been proposed that the kinds of vegetables that are usually consumed cooked (e.g., beans, peas, eggplant) may differ from those typically consumed raw (e.g., lettuce). Second, cooking processes can alter the digestibility of food as well as the bioavailability of nutrients (21). For example, Lee et al. found that vitamin C retention after cooking ranged from 0 to 91% for various combinations of cooking methods and vegetable, with higher retention after microwaving and lower retention after boiling (22). Third, the seasoning and oils used in cooking vegetables often increase intake of sodium and fat, which are known risk factors for CVD incidence and mortality (23, 24).

Despite these proposed mechanisms, this study indicates that observed associations of vegetable intakes with CVD risk and all-cause mortality are likely to be mostly accounted for by residual confounding. Studies using Mendelian randomization (which are less susceptible to confounding, and other biases of observational studies) might be particularly useful in reliably assessing the associations of diet on disease risk. For example, a recent Mendelian randomization study that used genetic determinants of plasma vitamin C concentration as a surrogate for vegetable intake reported a null association with ischemic heart disease (odds ratio 0.90 [0.75–1.08]) and all-cause mortality (odds ratio 0.88 [0.72–1.08]), despite strong inverse associations between vitamin C and these outcomes in observational analyses (25).

This study found the observed associations were mainly accounted for by socioeconomic status and lifestyle factors (26). Both the low socioeconomic status and major lifestyle factors, such as smoking and alcohol intake, are established risk factors for CVD, and there is strong evidence that the effect of socioeconomic status is partially mediated by the known lifestyle factors (27). For example, one study reported that an unhealthy lifestyle (including smoking, drinking, obesity, physical inactivity, and others) mediated 34–38% of the association between socioeconomic status and all-cause death (28). Therefore, given the complicated inter-relationship between socioeconomic status, lifestyle, and health outcomes, adjustment of measures of both socioeconomic status and lifestyle factors is likely to be important.

This study has some limitations. First, we did not measure intake of specific types of raw or cooked vegetables, nor were we able to account for differences in cooking methods. Second, vegetable intakes are self-reported in the baseline dietary questionnaire, although the validity and repeatability of the UK Biobank baseline dietary questionnaire have been evaluated and confirmed in previous studies (12). Third, we did not adjust for total calorie intake because such information was not available from the baseline dietary questionnaire, but we did adjusted for physical activity level and BMI, which has been shown as a valid method for isocaloric adjustment (29). Future studies should seek to address these limitations. However, such studies should also be aware of the importance of assessing reliably for residual confounding using similar methods to this report, or other approaches, such as Mendelian randomization.

Although this report does not find strong evidence of an association between higher vegetable intake and reduced risk of major CVD, the wider literature suggests that increasing vegetable intake is likely to reduce the risk of some other common diseases (4). As such, the public health recommendations on the benefits to health and the environment of a diet that is high in vegetable intake remain.

## Conclusion

In this study of 0.4 million middle-aged adults with 12-year follow-up, higher intakes of raw but not cooked vegetables were associated with lower CVD risk. However, given the large reductions in the predictive values of raw vegetable intake after adjustment for socioeconomic and lifestyle factors, residual confounding is likely to account for much, if not all, of the remaining associations. This study highlights the need for rigorous assessment for residual confounding in studies of the effects of diet and other lifestyle factors on disease risk and suggests the need to reappraise the evidence on the burden of CVD disease attributable to low vegetable intake in high-income populations.

## Data Availability Statement

Publicly available datasets were analyzed in this study. This data can be found here: https://www.ukbiobank.ac.uk/.

## Ethics Statement

The studies involving human participants were reviewed and UK Biobank was approved by the North West Multicenter Research Ethics Committee, the National Information Governance Board for Health and Social Care in England and Wales, and the Community Health Index Advisory Group in Scotland. The patients/participants provided their written informed consent to participate in this study.

## Author Contributions

QF designed the study and analyzed the data. QF, BL, JK, and MM interpreted results. QF drafted the manuscript. All the coauthors critically reviewed and revised the manuscript. All authors contributed to the article and approved the submitted version.

## Funding

This research was funded in whole, or part, by the Wellcome Trust [205339/Z/16/Z]. The funders had no role in the design and conduct of the study; collection, management, analysis, and interpretation of the data; preparation, review, or approval of the manuscript; or the decision to submit the manuscript for publication.

## Conflict of Interest

The authors declare that the research was conducted in the absence of any commercial or financial relationships that could be construed as a potential conflict of interest.

## Publisher's Note

All claims expressed in this article are solely those of the authors and do not necessarily represent those of their affiliated organizations, or those of the publisher, the editors and the reviewers. Any product that may be evaluated in this article, or claim that may be made by its manufacturer, is not guaranteed or endorsed by the publisher.
