# Supplementary material for: Raw and Cooked Vegetable Consumption and Risk of Cardiovascular Disease: A Study of 400,000 Adults in UK Biobank
Source: Front Nutr. 2022 Feb 21;9:831470. doi: 10.3389/fnut.2022.831470 (PMC8901125; doi:10.3389/fnut.2022.831470)
Supplement: Supplementary file 1 [file Data_Sheet_1.docx]

**Raw and Cooked Vegetable Consumptions with Cardiovascular Disease and Mortality: a Study of 400,000 Adults in UK Biobank**

**Supplementary file**

**Contents**

[Supplementary Methods 2](#_Toc92547310)

[Supplementary table 1: ICD codes for outcome ascertainment. 4](#_Toc92547311)

[Supplementary table 2: Baseline characteristics of eligible participants by their *total* vegetable consumption levels (number of heaped tablespoons/day) 5](#_Toc92547312)

[Supplementary table 3: Baseline characteristics of eligible participants by their *cooked* vegetable consumption levels (number of heaped tablespoons/day) 8](#_Toc92547313)

[Supplementary table 4: Baseline characteristics of eligible participants by their *raw* vegetable consumption levels (number of heaped tablespoons/day) 11](#_Toc92547314)

[Supplementary table 5: Associations between vegetable intake with CVD incidence, CVD mortality and all-cause mortality in models with progressive adjustment for potential confounders 14](#_Toc92547315)

[Supplementary table 6: Associations between raw/total vegetable proportion with incident CVD, incident MI, incident stroke, all-cause mortality, and CVD mortality, conditional on total vegetable intake. 17](#_Toc92547316)

[Supplementary table 7: Associations between vegetable intake with incident CVD, incident MI, incident stroke, all-cause mortality, and CVD mortality, after excluding the events occurring within two years after recruitment. 19](#_Toc92547317)

[Supplementary table 8: Associations between vegetable intake with incident CVD, incident MI, incident stroke, all-cause mortality, and CVD mortality in White population (n = 378 028). 21](#_Toc92547318)

[Supplementary table 9: Associations between vegetable intake with incident CVD, incident MI, incident stroke, all-cause mortality, and CVD mortality in non-White population (n = 21 558). 23](#_Toc92547319)

[Supplementary table 10: The basic characteristics and main findings of the previous studies investigating the independent effects of raw vs. cooked vegetable intake. 26](#_Toc92547320)

[Supplementary figure 1: the distributions of intakes of raw, cooked and total vegetable in UK Biobank cohort. (number of heaped tablespoons/day) 28](#_Toc92547321)

[Supplementary figure 2: Association of incident CVD with total, raw and cooked vegetable consumption 29](#_Toc92547322)

## Supplementary Methods

**Covariate measurement**

A range of covariates were considered in the statistical analyses, covering sociodemographic status, health status, medication use, and lifestyle factors. Sociodemographic characteristics included age, gender, ethnicity (White, Black, Asian, Mixed, Others), educational attainment (below secondary, lower secondary, upper secondary, vocational training, university) and Townsend deprivation index (an area-derived measure of socioeconomic inequality). Baseline health status included self-reported diagnosis of hypertension and diabetes, and use of antihypertensive drugs, statins and insulin.

Physical activity was assessed using the International Physical Activity Questionnaire and categorized into low, moderate, and high activity levels [1]. Briefly, high level of physical activity is defined as vigorous-intensity activity on at least 3 days achieving a minimum total physical activity of at least 1500 metabolic equivalent of task (MET)-minutes/week, or 7 days of any combination of walking, moderate-intensity or vigorous-intensity activities achieving a minimum total physical activity of at least 3000 MET-minutes/week; moderate level is defined as 3 or more days of vigorous-intensity activity of at least 20 minutes per day, or 5 or more days of moderate-intensity activity and/or walking of at least 30 minutes per day, or 5 or more days of any combination of walking, moderate-intensity or vigorous intensity activities achieving a minimum total physical activity of at least 600 MET-minutes/week; otherwise low level. Smoking and drinking status were coded as never, previous or current users. Body mass index (BMI) was calculated as body weight (kilograms) divided by height squared (meters).

Self-reported regular use of aspirin or ibuprofen, vitamin supplements (vitamin A, vitamin B, vitamin C, vitamin D, vitamin E, multi-vitamin, and folate) and mineral supplements (iron, calcium, selenium, zinc, and magnesium) were categorized as binary variables (yes/no). Frequency of intake of fresh fruit (0, 1 to 2, 3 to 4, ≥ 5 pieces/day), oily fish (< 1, 1, ≥ 2 times/week), non-oily fish (< 1, 1, ≥ 2 times/week), processed meat (0, ≤ 1, ≥ 2 times per week) and red meat were measured with baseline dietary questionnaire. Intake of red meat was the sum of pork, beef and mutton/lamb, and was transformed into a continuous variable using the method proposed by Bradbury et al. [2]. All answers of “unknown”, “do not know”, “prefer not to say” for each categorical variable were combined into one category of “unknown” and included in analyses as a separate category.

**Analysis on the likelihood ratio (LR) χ2 statistics**

We fitted a series of nested models with progressive adjustment for covariates. Model 1 (the basic model) was stratified by age group, gender, region and ethnicity, and only included the exposure of interest (vegetable intake). Based on Model 1, Model 2 was additionally adjusted for socioeconomic status (educational attainment, and Townsend deprivation index). Model 3 was based on Model 2, and additionally adjusted for lifestyle factors (physical activity, smoking, drinking, use of mineral supplements, use of vitamin supplements, fruit intake, oily fish intake, non-oily fish intake, red meat intake, processed meat intake). Model 4 was further adjusted for body mass index. Model 5 was further adjusted for baseline health status (hypertension, diabetes, use of antihypertensive drugs, use of statin, use of insulin, use of aspirin or ibuprofen), which was our analysis model.

We calculated the likelihood ratio (LR) χ2 statistic for vegetable intake (twice the increase in the log likelihood in models that do and do not include vegetable intake) in each model, which is a quantitative measure of the extent to which vegetable intake improves model fitting. Changes in LR χ2 statistic between the basic model and the model with covariate adjustment (Model 2, 3, 4, 5) are a measure of the extent to which the adjusted covariates account for the association between vegetable intake and health outcomes observed in the basic model. This method has been previously used in epidemiological studies [3,4]

**References**

1 World Health Organization. Global Physical Activity Questionnaire Analysis Guide. https://www.who.int/ncds/surveillance/steps/resources/GPAQ_Analysis_Guide.pdf

2 Bradbury KE, Young HJ, Guo W, *et al.* Dietary assessment in UK Biobank: an evaluation of the performance of the touchscreen dietary questionnaire. *J Nutr Sci* 2018;**7**:e6. doi:10.1017/jns.2017.66

3 Parish S, Peto R, Palmer A, *et al.* The joint effects of apolipoprotein B, apolipoprotein A1, LDL cholesterol, and HDL cholesterol on risk: 3510 cases of acute myocardial infarction and 9805 controls. *Eur Heart J* 2009;**30**:2137–46. doi:10.1093/eurheartj/ehp221

4 Floud S, Balkwill A, Moser K, *et al.* The role of health-related behavioural factors in accounting for inequalities in coronary heart disease risk by education and area deprivation: prospective study of 1.2 million UK women. *BMC Med* 2016;**14**:145. doi:10.1186/s12916-016-0687-2

## Supplementary table 1: ICD codes for outcome ascertainment.

| Outcomes | ICD9 codes | ICD10 codes |
| --- | --- | --- |
| CVD mortality | NA | I00-I99 |
| CVD incidence | 410, 410.0, 410.1, 410.2, 410.3, 410.4, 410.5, 410.6, 410.7, 410.8, 410.9  411, 411.0, 411.1, 411.8, 412.X,  429.79  430.X  431.X  434.X, 434.0, 434.1, 434.9  436.X | I21, I21.0, I21.1, I21.2, I21.3, I21.4, I21.9  I22, I22.0, I22.1, I22.8, I22.9  I23, I23.0, I23.1, I23.2, I23.3, I23.4, I23.5, I23.6, I23.8  I24.1  I25.2  I60, I60.0, I60.1, I60.2, I60.3, I60.4, I60.5, I60.6, I60.7, I60.8, I60.9  I61, I61.0, I61.1, I61.2, I61.3, I61.4, I61.5, I61.6, I61.8, I61.9  I63, I63.0, I63.1, I63.2, I63.3, I63.4, I63.5, I63.6, I63.8, I63.9  I64.X |
| MI incidence | 410, 410.0, 410.1, 410.2, 410.3, 410.4, 410.5, 410.6, 410.7, 410.8, 410.9  411, 411.0, 411.1, 411.8, 412.X,  429.79 | I21, I21.0, I21.1, I21.2, I21.3, I21.4, I21.9  I22, I22.0, I22.1, I22.8, I22.9  I23, I23.0, I23.1, I23.2, I23.3, I23.4, I23.5, I23.6, I23.8  I24.1  I25.2 |
| Stroke incidence | 430.X  431.X  434.X, 434.0, 434.1, 434.9  436.X | I60, I60.0, I60.1, I60.2, I60.3, I60.4, I60.5, I60.6, I60.7, I60.8, I60.9  I61, I61.0, I61.1, I61.2, I61.3, I61.4, I61.5, I61.6, I61.8, I61.9  I63, I63.0, I63.1, I63.2, I63.3, I63.4, I63.5, I63.6, I63.8, I63.9  I64.X |

Mortality outcomes were extracted from death registry, in which only ICD 10 codes are used. Incident outcomes were extracted from both death registry and inpatient databases, in which both ICD 9 and ICD 10 codes are used.

## Supplementary table 2: Baseline characteristics of eligible participants by their *total* vegetable consumption levels (number of heaped tablespoons/day)

|  | ≤1 tablespoon/day  (n=15 902) | 2-3 tablespoons/day  (n=109 536) | 4-7 tablespoons/day  (n=216 499) | ≥ 8 tablespoons/day  (n = 57 649) | All participants  (n=399 586) |
| --- | --- | --- | --- | --- | --- |
| Female (%) | 6 174 (38.83) | 54 948 (50.16) | 126 375 (58.37) | 33 997 (58.97) | 221 494 (55.43) |
| Age (years) | 53.96 (8.13) | 55.32 (8.16) | 56.52 (8.01) | 56.37 (7.99) | 56.07 (8.08) |
| Ethnicity (%) |  |  |  |  |  |
| White | 14 782 (93.25) | 104 731 (95.87) | 206 372 (95.61) | 52 143 (90.88) | 378 028 (94.91) |
| Black | 443 (2.79) | 1 509 (1.38) | 2 751 (1.27) | 1 235 (2.15) | 5 938 (1.49) |
| Asian | 361 (2.28) | 1 706 (1.56) | 3 978 (1.84) | 2 470 (4.30) | 8 515 (2.14) |
| Mixed/others | 266 (1.68) | 1 296 (1.19) | 2 743 (1.27) | 1530 (2.67) | 5 835 (1.46) |
| Townsend Deprivation index | -0.18 (3.51) | -1.43 (3.02) | -1.55 (2.93) | -1.13 (3.14) | -1.40 (3.03) |
| Education (%) |  |  |  |  |  |
| Below secondary | 3808 (24.45) | 15932 (14.76) | 31919 (14.98) | 9773 (17.32) | 61432 (15.63) |
| Lower secondary | 3329 (21.38) | 18420 (17.07) | 36146 (16.96) | 9043 (16.02) | 66938 (17.03) |
| Upper secondary | 850 (5.46) | 6339 (5.87) | 11861 (5.57) | 3095 (5.48) | 22145 (5.63) |
| Vocational | 4264 (27.38) | 30180 (27.97) | 59469 (27.9) | 15040 (26.65) | 108953 (27.72) |
| Higher education | 3321 (21.33) | 37040 (34.32) | 73733 (34.6) | 19483 (34.52) | 133577 (33.99) |
| Body mass index (kg/m^2^) | 28.00 (5.20) | 27.22 (4.70) | 27.27 (4.70) | 27.42 (4.82) | 27.31 (4.73) |
| Smoking (%) |  |  |  |  |  |
| Never | 7 759 (48.95) | 63 119 (57.77) | 121 274 (56.19) | 30 306 (52.79) | 222 458 (55.84) |
| Previous regular user | 4 608 (29.07) | 34 313 (31.40) | 75 145 (34.81) | 21 600 (37.62) | 135 666 (34.06) |
| Current regular user | 3 485 (21.98) | 11 828 (10.83) | 19 427 (9.00) | 5 506 (9.59) | 40 246 (10.1) |
| Drinking (%) |  |  |  |  |  |
| Never | 1 043 (6.57) | 4 118 (3.76) | 8 080 (3.73) | 3 169 (5.50) | 16 410 (4.11) |
| Previous regular user | 1 008 (6.35) | 3 463 (3.16) | 6 316 (2.92) | 2 303 (4.00) | 13 090 (3.28) |
| Current regular user | 13 817 (87.07) | 101 873 (93.07) | 201 964 (93.35) | 52 123 (90.50) | 369 777 (92.61) |
| Physical activity (%) |  |  |  |  |  |
| Low | 3 664 (29.70) | 19 926 (22.18) | 29 232 (16.44) | 6 158 (12.91) | 58 980 (18.00) |
| Moderate | 4 702 (38.11) | 38 452 (42.80) | 73 058 (41.09) | 17 195 (36.06) | 133 407 (40.71) |
| High | 3 971 (32.19) | 31 459 (35.02) | 75 528 (42.47) | 24 328 (51.02) | 135 286 (41.29) |
| Hypertension (%) | 4 172 (26.24) | 26 482 (24.18) | 55 071 (25.44) | 15 131 (26.25) | 100 856 (25.24) |
| Diabetes (%) | 994 (6.25) | 4 859 (4.44) | 9 904 (4.57) | 3 009 (5.22) | 18 766 (4.70) |
| Regular use of aspirin/ibuprofen (%) | 4 065 (25.56) | 26 039 (23.77) | 53 667 (24.79) | 14 394 (24.97) | 98 165 (24.57) |
| Regular use of mineral supplement (%) | 2 869 (18.04) | 25 789 (23.54) | 61 980 (28.63) | 17 955 (31.15) | 108 593 (27.18) |
| Regular use of vitamin supplement (%) | 1 760 (11.07) | 13 223 (12.07) | 30 534 (14.1) | 9 756 (16.92) | 55 273 (13.83) |
| Use of antihypertensive drugs (%) | 938 (5.90) | 8 133 (7.42) | 20 449 (9.45) | 5 628 (9.76) | 35 148 (8.80) |
| Use of statin (%) | 696 (4.38) | 5 426 (4.95) | 13 443 (6.21) | 3 822 (6.63) | 23 387 (5.85) |
| Use of insulin (%) | 58 (0.36) | 375 (0.34) | 855 (0.39) | 275 (0.48) | 1 563 (0.39) |
| Fruit intake (pieces/day) (%) |  |  |  |  |  |
| 0 | 5 102 (32.15) | 12 518 (11.44) | 12 738 (5.89) | 2 372 (4.12) | 32 730 (8.20) |
| 1 to 2 | 7 984 (50.32) | 67 783 (61.95) | 119 791 (55.39) | 23 572 (40.95) | 219 130 (54.90) |
| 3 to 4 | 2 098 (13.22) | 24 447 (22.35) | 67 970 (31.43) | 21 549 (37.43) | 116 064 (29.08) |
| ≥ 5 | 684 (4.31) | 4 659 (4.26) | 15 781 (7.30) | 10 076 (17.5) | 31 200 (7.82) |
| Oily fish intake (times/week) (%) |  |  |  |  |  |
| < 1/week | 11 293 (71.72) | 57 325 (52.52) | 84 863 (39.30) | 19 447 (33.85) | 172 928 (43.42) |
| 1/week | 1 185 (7.53) | 12 479 (11.43) | 42 534 (19.70) | 16 317 (28.40) | 152 798 (38.37) |
| > 1/week | 3 267 (20.75) | 39 335 (36.04) | 88 514 (41.00) | 21 682 (37.74) | 72 515 (18.21) |
| Non-oily fish intake (times/week) (%) |  |  |  |  |  |
| <1/week | 8 665 (54.95) | 41 288 (37.79) | 65 661 (30.40) | 16 570 (28.83) | 132 184 (33.17) |
| 1/week | 5 539 (35.13) | 54 524 (49.91) | 112 771 (52.20) | 27 208 (47.34) | 200 042 (50.20) |
| >1/week | 1 565 (9.92) | 13 441 (12.3) | 37 587 (17.40) | 13 698 (23.83) | 66 291(16.63) |
| Processed meat intake (times/week) (%) |  |  |  |  |  |
| Never | 1 018 (6.42) | 6 956 (6.36) | 20 619 (9.53) | 9 643 (16.75) | 38 236 (9.58) |
| ≤ 1 per week | 7 900 (49.79) | 64 169 (58.63) | 133 449 (61.68) | 33 808 (58.71) | 239 326 (59.94) |
| ≥ 2 per week | 6 949 (43.80) | 38 331 (35.02) | 62 278 (28.79) | 14 132 (24.54) | 121 690 (30.48) |
| Red meat intake (times/week) | 2.04 (1.59) | 2.15 (1.39) | 2.12 (1.42) | 2.03 (1.58) | 2.11 (1.44) |
| Cooked vegetable intake (tablespoons/day) (%) |  |  |  |  |  |
| 0 | 7 606 (47.83) | 2 296 (2.10) | 1 660 (0.77) | 540 (0.94) | 12 102 (3.03) |
| 1 to 2 | 8 296 (52.17) | 99 670 (90.99) | 77 120 (35.62) | 3 841 (6.66) | 188 927 (47.28) |
| 3 to 4 | 0 (0.00) | 7 570 (6.91) | 126 290 (58.33) | 22 659 (39.31) | 156 519 (39.17) |
| ≥5 | 0 (0.00) | 0 (0.00) | 11 429 (5.28) | 30 609 (53.10) | 42038 (10.52) |
| Cooked vegetable intake (tablespoons/day) | 0.52 (0.50) | 1.69 (0.63) | 2.84 (0.96) | 5.26 (3.38) | 2.78 (1.92) |
| Raw vegetable intake (tablespoons/day) (%) |  |  |  |  |  |
| 0 | 13 600 (85.52) | 20 546 (18.76) | 4 788 (2.21) | 502 (0.87) | 39 436 (9.87) |
| 1 to 2 | 2 302 (14.48) | 88 078 (80.41) | 134 440 (62.1) | 4 341 (7.53) | 229 161 (57.35) |
| 3 to 4 | 0 (0.00) | 912 (0.83) | 70 592 (32.61) | 18 633 (32.32) | 90 137 (22.56) |
| ≥5 | 0 (0.00) | 0 (0.00) | 6 679 (3.09) | 34 173 (59.28) | 40 852 (10.22) |
| Raw vegetable intake (tablespoons/day) | 0.14 (0.35) | 0.93 (0.56) | 2.25 (1.07) | 5.47 (3.51) | 2.27 (2.15) |
| Total vegetable intake (tablespoons/day) | 0.67 (0.47) | 2.63 (0.48) | 5.09 (1.04) | 10.73 (5.03) | 5.05 (3.37) |

Mean (SD) for continuous variables and frequency (percentage) for categorical variables

## Supplementary table 3: Baseline characteristics of eligible participants by their *cooked* vegetable consumption levels (number of heaped tablespoons/day)

|  | <1 tablespoon/day  (n=12 102) | 1-2 tablespoons/day  (n=188 927) | 3-4 tablespoons/day  (n=156 519) | ≥ 5 tablespoons/day  (n = 42 038) | All participants (n=399 586) |
| --- | --- | --- | --- | --- | --- |
| Female (%) | 5 506 (45.50) | 104 695 (55.42) | 89 881 (57.42) | 21 412 (50.93) | 221 494 (55.43) |
| Age (years) | 53.48 (8.00) | 55.37 (8.09) | 56.97 (7.96) | 56.6 (8.11) | 56.07 (8.08) |
| Ethnicity (%) |  |  |  |  |  |
| White | 10 951 (90.77) | 180 574 (95.83) | 149 084 (95.58) | 37 419 (89.44) | 378 028 (94.91) |
| Black | 396 (3.28) | 2 569 (1.36) | 1 998 (1.28) | 975 (2.33) | 5 938 (1.49) |
| Asian | 431 (3.57) | 2 879 (1.53) | 2 904 (1.86) | 2 301 (5.5) | 8 515 (2.14) |
| Mixed/others | 287 (2.38) | 2 412 (1.28) | 1 995 (1.28) | 1 141 (2.73) | 5 835 (1.46) |
| Townsend Deprivation index | 0.25 (3.57) | -1.46 (3) | -1.56 (2.92) | -1.05 (3.18) | -1.4 (3.03) |
| Education (%) |  |  |  |  |  |
| Lower than secondary | 3045 (25.77) | 25850 (13.9) | 25178 (16.34) | 7359 (17.88) | 61432 (15.63) |
| Lower secondary | 2499 (21.15) | 32228 (17.33) | 26022 (16.89) | 6189 (15.04) | 66938 (17.03) |
| Upper secondary | 639 (5.41) | 11041 (5.94) | 8271 (5.37) | 2194 (5.33) | 22145 (5.63) |
| Vocational | 3072 (26.00) | 51314 (27.59) | 43566 (28.28) | 11001 (26.73) | 108953 (27.72) |
| Higher education | 2559 (21.66) | 65572 (35.25) | 51040 (33.13) | 14406 (35.01) | 133577 (33.99) |
| Body mass index (kg/m^2^) | 27.98 (5.40) | 27.12 (4.67) | 27.41 (4.74) | 27.56 (4.81) | 27.31 (4.73) |
| Smoking (%) |  |  |  |  |  |
| Never | 5 567 (46.18) | 108 813 (57.74) | 86 193 (55.25) | 21 885 (52.28) | 222 458 (55.84) |
| Previous regular user | 3 642 (30.21) | 60 490 (32.10) | 55 762 (35.74) | 15 772 (37.68) | 135 666 (34.06) |
| Current regular user | 2 847 (23.61) | 19 140 (10.16) | 14 057 (9.01) | 4 202 (10.04) | 40 246 (10.10) |
| Drinking (%) |  |  |  |  |  |
| Never | 939 (7.78) | 6 802 (3.60) | 6 137 (3.92) | 2 532 (6.03) | 16 410 (4.11) |
| Previous regular user | 898 (7.44) | 5 512 (2.92) | 4 870 (3.11) | 1 810 (4.31) | 13 090 (3.28) |
| Current regular user | 10 235 (84.78) | 176 483 (93.48) | 145 406 (92.96) | 37 653 (89.66) | 369 777 (92.61) |
| Physical activity (%) |  |  |  |  |  |
| Low | 2 500 (26.70) | 31 147 (20.12) | 20 490 (15.95) | 4 843 (13.81) | 58 980 (18.00) |
| Moderate | 3 431 (36.63) | 65 703 (42.44) | 51 569 (40.15) | 12 704 (36.22) | 133 407 (40.71) |
| High | 3 434 (36.67) | 57 960 (37.44) | 56 368 (43.89) | 17 524 (49.97) | 135 286 (41.29) |
| Hypertension (%) | 3 147 (26.00) | 44 835 (23.73) | 41 429 (26.47) | 11 445 (27.23) | 100 856 (25.24) |
| Diabetes (%) | 742 (6.13) | 7 872 (4.17) | 7 647 (4.89) | 2 505 (5.96) | 18 766 (4.7) |
| Regular use of aspirin/ibuprofen (%) | 3 192 (26.38) | 45 246 (23.95) | 39 075 (24.97) | 10 652 (25.34) | 98 165 (24.57) |
| Regular use of mineral supplement (%) | 2 397 (19.81) | 48 041 (25.43) | 45 810 (29.27) | 12 345 (29.37) | 108 593 (27.18) |
| Regular use of vitamin supplement (%) | 1 523 (12.58) | 24 302 (12.86) | 22 593 (14.43) | 6 855 (16.31) | 55 273 (13.83) |
| Use of antihypertensive drugs (%) | 805 (6.65) | 15 220 (8.06) | 15 428 (9.86) | 3 695 (8.79) | 35 148 (8.80) |
| Use of statin (%) | 579 (4.78) | 9 853 (5.22) | 10 425 (6.66) | 2 530 (6.02) | 23 387 (5.85) |
| Use of insulin (%) | 61 (0.50) | 649 (0.34) | 672 (0.43) | 181 (0.43) | 1 563 (0.39) |
| Fruit intake (pieces/day) (%) |  |  |  |  |  |
| 0 | 3 428 (28.41) | 17 617 (9.34) | 9 353 (5.98) | 2 332 (5.56) | 32 730 (8.20) |
| 1 to 2 | 5 429 (44.99) | 112 159 (59.43) | 83 213 (53.22) | 18 329 (43.66) | 219 130 (54.90) |
| 3 to 4 | 2 174 (18.02) | 48 541 (25.72) | 50 896 (32.55) | 14 453 (34.43) | 116 064 (29.08) |
| ≥ 5 | 1 036 (8.59) | 10 401 (5.51) | 12 899 (8.25) | 6 864 (16.35) | 31 200 (7.82) |
| Oily fish intake (times/week) (%) |  |  |  |  |  |
| < 1/week | 8 161 (68.17) | 90 353 (47.98) | 59 175 (37.92) | 15 239 (36.39) | 172 928 (43.42) |
| 1/week | 2 466 (20.60) | 70 954 (37.68) | 63 723 (40.83) | 15 655 (37.38) | 152 798 (38.37) |
| > 1/week | 1 344 (11.23) | 27 018 (14.35) | 33 165 (21.25) | 10 988 (26.24) | 72 515 (18.21) |
| Non-oily fish intake (times/week) (%) |  |  |  |  |  |
| <1/week | 6 582 (54.94) | 66 128 (35.08) | 46 432 (29.74) | 13 042 (31.12) | 132 184 (33.17) |
| 1/week | 3 810 (31.80) | 95 131 (50.47) | 81 385 (52.12) | 19 716 (47.05) | 200 042 (50.20) |
| >1/week | 1 589 (13.26) | 27 224 (14.44) | 28 330 (18.14) | 9 148 (21.83) | 66 291 (16.63) |
| Processed meat intake (times/week) (%) |  |  |  |  |  |
| Never | 1 275 (10.56) | 13 926 (7.38) | 15 802 (10.10) | 7 233 (17.23) | 38 236 (9.58) |
| ≤ 1/week | 5 956 (49.34) | 112 894 (59.8) | 96 591 (61.75) | 23 885 (56.89) | 239 326 (59.94) |
| ≥ 2/week | 4 841 (40.10) | 61 962 (32.82) | 44 020 (28.14) | 10 867 (25.88) | 121 690 (30.48) |
| Red meat intake (times/week) | 1.78 (1.58) | 2.08 (1.35) | 2.17 (1.46) | 2.12 (1.68) | 2.11 (1.44) |
| Total vegetable intake (tablespoons/day) (%) |  |  |  |  |  |
| ≤1 | 7 606 (62.85) | 8 296 (4.39) | 0 (0.00) | 0 (0.00) | 15 902 (3.98) |
| 2 to 3 | 2 296 (18.97) | 99 670 (52.76) | 7 570 (4.84) | 0 (0.00) | 109 536 (27.41) |
| 4 to 7 | 1 660 (13.72) | 77 120 (40.82) | 126 290 (80.69) | 11 429 (27.19) | 216 499 (54.18) |
| ≥8 | 540 (4.46) | 3 841 (2.03) | 22 659 (14.48) | 30 609 (72.81) | 57 649 (14.43) |
| Cooked vegetable intake (tablespoons/day) | 0 (0) | 1.71 (0.45) | 3.29 (0.45) | 6.51 (3.34) | 2.78 (1.92) |
| Raw vegetable intake (tablespoons/day) (%) |  |  |  |  |  |
| 0 | 5 304 (43.83) | 21 272 (11.26) | 10 371 (6.63) | 2 489 (5.92) | 39 436 (9.87) |
| 1 to 2 | 3 686 (30.46) | 128 028 (67.77) | 83 664 (53.45) | 13 783 (32.79) | 229 161 (57.35) |
| 3 to 4 | 1 528 (12.63) | 30 151 (15.96) | 47 603 (30.41) | 10 855 (25.82) | 90 137 (22.56) |
| ≥5 | 1 584 (13.09) | 9 476 (5.02) | 14 881 (9.51) | 14 911 (35.47) | 40 852 (10.22) |
| Raw vegetable intake (tablespoons/day) | 1.87 (3.11) | 1.8 (1.59) | 2.4 (1.81) | 4 (3.68) | 2.27 (2.15) |
| Total vegetable intake(tablespoons/day) | 1.87 (3.11) | 3.51 (1.69) | 5.69 (1.92) | 10.51 (5.79) | 5.05 (3.37) |

Mean (SD) for continuous variables and frequency (percentage) for categorical variables.

## Supplementary table 4: Baseline characteristics of eligible participants by their *raw* vegetable consumption levels (number of heaped tablespoons/day)

|  | <1 tablespoon/day  (n=39 436) | 1-2 tablespoons/day (n=229 161) | 3-4 tablespoons/day (n=90 137) | ≥ 5 tablespoons/day (n = 40 852) | All participants (n=399 586) |
| --- | --- | --- | --- | --- | --- |
| Female (%) | 15736 (39.9) | 123145 (53.74) | 56831 (63.05) | 25782 (63.11) | 221494 (55.43) |
| Age (years) | 56.07 (8.25) | 55.96 (8.12) | 56.49 (7.96) | 55.79 (7.94) | 56.07 (8.08) |
| Ethnicity (%) |  |  |  |  |  |
| White | 37 163 (94.56) | 219 012 (95.84) | 84 857 (94.47) | 36 996 (90.96) | 378 028 (94.91) |
| Black | 747 (1.90) | 2 842 (1.24) | 1 438 (1.60) | 911 (2.24) | 5 938 (1.49) |
| Asian | 922 (2.35) | 3 940 (1.72) | 2 058 (2.29) | 1 595 (3.92) | 8 515 (2.14) |
| Mixed/others | 468 (1.19) | 2 723 (1.19) | 1 471 (1.64) | 1 173 (2.88) | 5 835 (1.46) |
| Townsend Deprivation index | -0.8 (3.31) | -1.56 (2.94) | -1.41 (3.00) | -1.07 (3.18) | -1.4 (3.03) |
| Education (%) |  |  |  |  |  |
| Lower than secondary | 9383 (24.25) | 31166 (13.80) | 14362 (16.22) | 6521 (16.32) | 61432 (15.63) |
| Lower secondary | 7403 (19.13) | 37518 (16.61) | 15423 (17.41) | 6594 (16.5) | 66938 (17.03) |
| Upper secondary | 2157 (5.57) | 12923 (5.72) | 4803 (5.42) | 2262 (5.66) | 22145 (5.63) |
| Vocational | 10808 (27.93) | 63373 (28.06) | 24152 (27.27) | 10620 (26.57) | 108953 (27.72) |
| Higher education | 8946 (23.12) | 80832 (35.8) | 29827 (33.68) | 13972 (34.96) | 133577 (33.99) |
| Body mass index (kg/m^2^) | 27.77 (4.98) | 27.25 (4.67) | 27.28 (4.76) | 27.27 (4.82) | 27.31 (4.73) |
| Smoking (%) |  |  |  |  |  |
| Never | 20 250 (51.51) | 130 861 (57.26) | 50 025 (55.68) | 21 322 (52.40) | 222 458 (55.84) |
| Previous regular user | 12 471 (31.72) | 76 139 (33.32) | 31 714 (35.30) | 15 342 (37.70) | 135 666 (34.06) |
| Current regular user | 6 595 (16.77) | 21 522 (9.42) | 8 102 (9.02) | 4 027 (9.90) | 40 246 (10.10) |
| Drinking (%) |  |  |  |  |  |
| Never | 2 334 (5.93) | 8 116 (3.54) | 3 678 (4.08) | 2 282 (5.59) | 16 410 (4.11) |
| Previous regular user | 2 111 (5.36) | 6 544 (2.86) | 2 856 (3.17) | 1 579 (3.87) | 13 090 (3.28) |
| Current regular user | 34 936 (88.71) | 214 356 (93.6) | 83 532 (92.75) | 36 953 (90.54) | 369 777 (92.61) |
| Physical activity (%) |  |  |  |  |  |
| Low | 8 097 (26.12) | 35 741 (18.88) | 10 727 (14.56) | 4 415 (13.11) | 58 980 (18) |
| Moderate | 12 135 (39.15) | 79 896 (42.20) | 29 165 (39.58) | 12 211 (36.25) | 133 407 (40.71) |
| High | 10 763 (34.72) | 73 668 (38.91) | 33 798 (45.87) | 17 057 (50.64) | 135 286 (41.29) |
| Hypertension (%) | 10 740 (27.23) | 56 863 (24.81) | 22 984 (25.50) | 10 269 (25.14) | 100 856 (25.24) |
| Diabetes (%) | 2 468 (6.260) | 10 200 (4.45) | 4 113 (4.56) | 1 985 (4.86) | 18 766 (4.70) |
| Regular use of aspirin/ibuprofen (%) | 9 803 (24.86) | 55 809 (24.35) | 22 402 (24.85) | 10 151 (24.85) | 98 165 (24.57) |
| Regular use of mineral supplement (%) | 8 358 (21.19) | 60 260 (26.30) | 27 106 (30.07) | 12 869 (31.50) | 108 593 (27.18) |
| Regular use of vitamin supplement (%) | 4 681 (11.87) | 29 972 (13.08) | 13 552 (15.03) | 7 068 (17.30) | 55 273 (13.83) |
| Use of antihypertensive drugs (%) | 2 700 (6.85) | 19 290 (8.42) | 9 171 (10.17) | 3 987 (9.76) | 35 148 (8.80) |
| Use of statin (%) | 2 020 (5.12) | 12 689 (5.54) | 6 103 (6.77) | 2 575 (6.30) | 23 387 (5.85) |
| Use of insulin (%) | 162 (0.41) | 810 (0.35) | 384 (0.43) | 207 (0.51) | 1 563 (0.39) |
| Fruit intake (pieces/day) (%) |  |  |  |  |  |
| 0 | 9 161 (23.27) | 17 844 (7.79) | 4 114 (4.57) | 1 611 (3.95) | 32 730 (8.20) |
| 1 to 2 | 21 237 (53.95) | 136 881 (59.79) | 44 863 (49.83) | 16 149 (39.58) | 219 130 (54.90) |
| 3 to 4 | 7 095 (18.02) | 61 576 (26.90) | 32 212 (35.77) | 15 181 (37.21) | 116 064 (29.08) |
| ≥ 5 | 1 872 (4.76) | 12 619 (5.51) | 8 852 (9.83) | 7 857 (19.26) | 31 200 (7.82) |
| Oily fish intake (times/week) (%) |  |  |  |  |  |
| < 1/week | 24 530 (62.67) | 101 835 (44.57) | 32 556 (36.22) | 14 007 (34.42) | 172 928 (43.42) |
| 1/week | 10 709 (27.36) | 90 846 (39.76) | 36 205 (40.28) | 15 038 (36.95) | 152 798 (38.37) |
| > 1/week | 3 905 (9.98) | 35 823 (15.68) | 21 133 (23.51) | 11 654 (28.63) | 72 515 (18.21) |
| Non-oily fish intake (times/week) (%) |  |  |  |  |  |
| <1/week | 18 430 (46.99) | 76 145 (33.30) | 25 671 (28.55) | 11 938 (29.31) | 132184 (33.17) |
| 1/week | 16 514 (42.11) | 118 895 (52.00) | 45 869 (51.01) | 18 764 (46.07) | 200042 (50.20) |
| >1/week | 4 274 (10.90) | 33 613 (14.70) | 18 378 (20.44) | 10 026 (24.62) | 66291 (16.63) |
| Processed meat intake (times/week) (%) |  |  |  |  |  |
| Never | 2 785 (7.07) | 18 177 (7.94) | 10 334 (11.47) | 6 940 (17.01) | 38 236 (9.58) |
| ≤ 1 per week | 21 359 (54.25) | 138 108 (60.30) | 55 675 (61.82) | 24 184 (59.28) | 239 326 (59.94) |
| ≥ 2 per week | 15 228 (38.68) | 72 733 (31.76) | 24 056 (26.71) | 9 673 (23.71) | 121 690 (30.48) |
| Red meat intake (times/week) | 2.25 (1.61) | 2.14 (1.4) | 2.04 (1.42) | 1.94 (1.53) | 2.11 (1.44) |
| Cooked vegetable intake (tablespoons/day) (%) |  |  |  |  |  |
| 0 | 5 304 (13.45) | 3 686 (1.61) | 1 528 (1.70) | 1 584 (3.88) | 12 102 (3.03) |
| 1 to 2 | 21 272 (53.94) | 128 028 (55.87) | 30 151 (33.45) | 9 476 (23.20) | 188 927 (47.28) |
| 3 to 4 | 10 371 (26.30) | 83 664 (36.51) | 47 603 (52.81) | 14 881 (36.43) | 156 519 (39.17) |
| ≥5 | 2 489 (6.31) | 13 783 (6.01) | 10 855 (12.04) | 14 911 (36.50) | 42 038 (10.52) |
| Cooked vegetable intake (tablespoons/day) | 2.14 (1.76) | 2.51 (1.42) | 3.08 (1.73) | 4.27 (1.73) | 2.78 (1.92) |
| Total vegetable intake (tablespoons/day) (%) |  |  |  |  |  |
| ≤1 | 13 600 (34.49) | 2 302 (1.00) | 0 (0.00) | 0 (0.00) | 15 902 (3.98) |
| 2 to 3 | 20 546 (52.1) | 88 078 (38.43) | 912 (1.01) | 0 (0.00) | 109 536 (27.41) |
| 4 to 7 | 4 788 (12.14) | 134 440 (58.67) | 70 592 (78.32) | 6 679 (16.35) | 216 499 (54.18) |
| ≥8 | 502 (1.27) | 4 341 (1.89) | 18 633 (20.67) | 34 173 (83.65) | 57 649 (14.43) |
| Raw vegetable intake (tablespoons/day) | 0 (0.00) | 1.45 (0.5) | 3.34 (0.47) | 6.71 (3.53) | 2.27 (2.15) |
| Total vegetable intake(tablespoons/day) | 2.14 (1.76) | 3.96 (1.57) | 6.42 (1.84) | 10.98 (5.7) | 5.05 (3.37) |

Mean (SD) for continuous variables and frequency (percentage) for categorical variables.

## Supplementary table 5: Associations between vegetable intake with CVD incidence, CVD mortality and all-cause mortality in models with progressive adjustment for potential confounders

|  | HR (95%CI)* | LR χ2 statistic | Reduction (%) in LR χ2 statistic‡ |
| --- | --- | --- | --- |
| **CVD incidence** | |  |  |
| Total vegetable intake | |  |  |
| (1): Basic model | 0.74 (0.69, 0.80) | 87.8 |  |
| (2): (1) + SES | 0.80 (0.74, 0.86) | 45.8 | 47.9 |
| (3): (2) + Lifestyle factors | 0.90 (0.83, 0.97) | 11.1 | 87.4 |
| (4): (3) + BMI | 0.89 (0.83, 0.97) | 11.0 | 87.4 |
| (5): (4) + health status | 0.90 (0.83, 0.97) | 10.1 | 88.6 |
| Raw vegetable intake | |  |  |
| (1): Basic model | 0.79 (0.74, 0.84) | 127.9 |  |
| (2): (1) + SES | 0.83 (0.78, 0.88) | 72.6 | 43.2 |
| (3): (2) + Lifestyle factors † | 0.88 (0.82, 0.94) | 24.3 | 81.0 |
| (4): (3) + BMI † | 0.88 (0.82, 0.94) | 25.3 | 80.3 |
| (5): (4) + health status † | 0.88 (0.83, 0.94) | 23.2 | 81.9 |
| Cooked vegetable intake | |  |  |
| (1): Basic model | 0.77 (0.71, 0.84) | 53.0 |  |
| (2): (1) + SES | 0.85 (0.78, 0.93) | 21.7 | 59.0 |
| (3): (2) + Lifestyle factors † | 1.00 (0.91, 1.09) | 5.3 | 90.0 |
| (4): (3) + BMI † | 1.00 (0.91, 1.09) | 2.3 | 95.8 |
| (5): (4) + health status † | 1.00 (0.91, 1.09) | 1.5 | 97.2 |
|  | |  |  |
| **MI incidence** | |  |  |
| Total vegetable intake | |  |  |
| (1): Basic model | 0.71 (0.65, 0.78) | 75.1 |  |
| (2): (1) + SES | 0.77 (0.70, 0.84) | 41.8 | 44.3 |
| (3): (2) + Lifestyle factors | 0.87 (0.79, 0.95) | 12.3 | 83.6 |
| (4): (3) + BMI | 0.86 (0.78, 0.95) | 12.0 | 84.0 |
| (5): (4) + health status | 0.87 (0.79, 0.95) | 11.1 | 85.2 |
| Raw vegetable intake | |  |  |
| (1): Basic model | 0.78 (0.72, 0.84) | 88.8 |  |
| (2): (1) + SES | 0.82 (0.75, 0.88) | 49.2 | 44.6 |
| (3): (2) + Lifestyle factors † | 0.88 (0.81, 0.96) | 14.0 | 84.2 |
| (4): (3) + BMI † | 0.88 (0.81, 0.95) | 14.6 | 83.6 |
| (5): (4) + health status † | 0.88 (0.81, 0.96) | 13.3 | 85.0 |
| Cooked vegetable intake | |  |  |
| (1): Basic model | 0.74 (0.67, 0.83) | 42.8 |  |
| (2): (1) + SES | 0.82 (0.73, 0.91) | 18.6 | 56.7 |
| (3): (2) + Lifestyle factors † | 0.96 (0.86, 1.08) | 2.5 | 94.2 |
| (4): (3) + BMI † | 0.97 (0.86, 1.08) | 0.9 | 98.0 |
| (5): (4) + health status † | 0.97 (0.86, 1.08) | 0.6 | 98.5 |
|  | |  |  |
|  | |  |  |
| **Stroke incidence** | |  |  |
| Total vegetable intake | |  |  |
| (1): Basic model | 0.84 (0.74, 0.95) | 18.8 |  |
| (2): (1) + SES | 0.90 (0.79, 1.02) | 8.6 | 54.4 |
| (3): (2) + Lifestyle factors | 1.01 (0.89, 1.15) | 2.6 | 86.2 |
| (4): (3) + BMI | 1.00 (0.88, 1.14) | 2.3 | 88.0 |
| (5): (4) + health status | 1.01 (0.89, 1.15) | 2.2 | 88.1 |
| Raw vegetable intake | |  |  |
| (1): Basic model | 0.85 (0.77, 0.94) | 31.7 |  |
| (2): (1) + SES | 0.89 (0.80, 0.98) | 17.2 | 45.9 |
| (3): (2) + Lifestyle factors † | 0.91 (0.82, 1.02) | 6.1 | 80.7 |
| (4): (3) + BMI † | 0.91 (0.82, 1.02) | 6.3 | 80.1 |
| (5): (4) + health status † | 0.92 (0.83, 1.02) | 5.6 | 82.2 |
| Cooked vegetable intake | |  |  |
| (1): Basic model | 0.87 (0.75, 1.01) | 19.1 |  |
| (2): (1) + SES | 0.95 (0.82, 1.10) | 9.9 | 48.3 |
| (3): (2) + Lifestyle factors † | 1.09 (0.94, 1.27) | 7.9 | 58.5 |
| (4): (3) + BMI † | 1.09 (0.94, 1.27) | 6.5 | 66.1 |
| (5): (4) + health status † | 1.09 (0.94, 1.27) | 6.1 | 68.3 |
|  | |  |  |
| **CVD mortality** | |  |  |
| Total vegetable intake | |  |  |
| (1): Basic model | 0.63 (0.55, 0.73) | 58.2 |  |
| (2): (1) + SES | 0.71 (0.61, 0.81) | 32.2 | 44.7 |
| (3): (2) + Lifestyle factors | 0.83 (0.72, 0.96) | 8.8 | 84.8 |
| (4): (3) + BMI | 0.82 (0.71, 0.95) | 8.8 | 84.9 |
| (5): (4) + health status | 0.83 (0.71, 0.96) | 8.0 | 86.3 |
| Raw vegetable intake | |  |  |
| (1): Basic model | 0.74 (0.65, 0.84) | 63.8 |  |
| (2): (1) + SES | 0.79 (0.70, 0.89) | 34.2 | 46.3 |
| (3): (2) + Lifestyle factors † | 0.85 (0.74, 0.97) | 8.8 | 86.2 |
| (4): (3) + BMI † | 0.84 (0.74, 0.97) | 9.0 | 86.0 |
| (5): (4) + health status † | 0.85 (0.74, 0.97) | 8.2 | 87.2 |
| Cooked vegetable intake | |  |  |
| (1): Basic model | 0.67 (0.57, 0.78) | 53.9 |  |
| (2): (1) + SES | 0.77 (0.65, 0.90) | 28.1 | 47.9 |
| (3): (2) + Lifestyle factors † | 0.96 (0.80, 1.13) | 8.6 | 84.1 |
| (4): (3) + BMI † | 0.96 (0.80, 1.13) | 6.6 | 87.8 |
| (5): (4) + health status † | 0.96 (0.80, 1.13) | 6.3 | 88.4 |
|  | |  |  |
| **All-cause mortality** | |  |  |
| Total vegetable intake | |  |  |
| (1): Basic model | 0.61 (0.57, 0.65) | 298.7 |  |
| (2): (1) + SES | 0.67 (0.63, 0.72) | 180.7 | 39.5 |
| (3): (2) + Lifestyle factors | 0.79 (0.74, 0.85) | 61.3 | 79.5 |
| (4): (3) + BMI | 0.79 (0.74, 0.84) | 61.4 | 79.5 |
| (5): (4) + health status | 0.80 (0.74, 0.85) | 57.9 | 80.6 |
| Raw vegetable intake | |  |  |
| (1): Basic model | 0.69 (0.65, 0.73) | 352.7 |  |
| (2): (1) + SES | 0.73 (0.69, 0.77) | 216.9 | 38.5 |
| (3): (2) + Lifestyle factors † | 0.81 (0.76, 0.86) | 61.7 | 82.5 |
| (4): (3) + BMI † | 0.81 (0.76, 0.86) | 62.0 | 82.4 |
| (5): (4) + health status † | 0.82 (0.77, 0.87) | 57.3 | 83.8 |
| Cooked vegetable intake | |  |  |
| (1): Basic model | 0.57 (0.53, 0.61) | 347.8 |  |
| (2): (1) + SES | 0.64 (0.60, 0.69) | 212.5 | 38.9 |
| (3): (2) + Lifestyle factors † | 0.81 (0.75, 0.87) | 77.6 | 77.7 |
| (4): (3) + BMI † | 0.81 (0.75, 0.87) | 72.9 | 79.1 |
| (5): (4) + health status † | 0.81 (0.75, 0.87) | 72.0 | 79.3 |

*Hazard ratios (HR) for highest vs lowest (reference) intake. HR in the basic model were stratified by age group, gender, ethnicity and region, and progressively adjusted for other factors, where indicated, such that the final model includes all the listed covariates for socioeconomic, lifestyle and health status combined (see Supplementary Methods).

†: Raw vegetable intake and cooked vegetable intake were mutually adjusted for in models.

‡: Change (%) in LR χ2 statistic, compared to the basic model. AIC: Akaika information criterion.

## Supplementary table 6: Associations between raw/total vegetable proportion with incident CVD, incident MI, incident stroke, all-cause mortality, and CVD mortality, conditional on total vegetable intake.

|  | **Incident CVD** | | |  | **Incident MI** | | |  | **Incident stroke** | | |
| --- | --- | --- | --- | --- | --- | --- | --- | --- | --- | --- | --- |
|  | Cases/total | HR (95%CI) | p |  | Cases/total | HR (95%CI) | p |  | Cases/total | HR (95%CI) | p |
| Total vegetable |  |  |  |  |  |  |  |  |  |  |  |
| 0-1 | 612 /10475 | Reference |  |  | 417 /10497 | Reference |  |  | 210 /10579 | Reference |  |
| 2-3 | 4974 /108479 | 0.93 (0.86, 1.02) | 0.111 |  | 3141 /108652 | 0.89 (0.80, 0.99) | 0.033 |  | 1889 /109391 | 0.99 (0.86, 1.15) | 0.916 |
| 4-7 | 9509 /214493 | 0.92 (0.84, 0.99) | 0.044 |  | 5899 /214824 | 0.88 (0.79, 0.97) | 0.013 |  | 3710 /216217 | 0.98 (0.85, 1.14) | 0.820 |
| >7 | 2599 /57083 | 0.93 (0.85, 1.02) | 0.143 |  | 1617 /57184 | 0.88 (0.79, 0.99) | 0.034 |  | 1044 /57558 | 1.05 (0.90, 1.23) | 0.538 |
| raw/total ratio |  |  |  |  |  |  |  |  |  |  |  |
| <0.33 | 4888 /90727 | Reference |  |  | 3125 /90891 | Reference |  |  | 1845 /91650 | Reference |  |
| 0.33-0.42 | 4846 /106939 | 0.97 (0.93, 1.01) | 0.146 |  | 3000 /107101 | 0.97 (0.92, 1.02) | 0.216 |  | 1922 /107826 | 0.99 (0.92, 1.06) | 0.724 |
| 0.43-0.50 | 4734 /109984 | 0.96 (0.92, 1.01) | 0.063 |  | 2938 /110160 | 0.96 (0.91, 1.01) | 0.151 |  | 1825 /110837 | 0.95 (0.89, 1.02) | 0.154 |
| >0.50 | 3226 /82880 | 0.93 (0.89, 0.98) | 0.004 |  | 2011 /83005 | 0.95 (0.90, 1.01) | 0.121 |  | 1261 /83432 | 0.92 (0.85, 0.99) | 0.021 |
|  |  |  |  |  |  |  |  |  |  |  |  |
|  | **All-cause mortality** | | |  | **CVD mortality** | | |  |  | | |
|  | Cases/total | HR (95%CI) | p |  | Cases/total | HR (95%CI) | p |  |  |  |  |
| Total vegetable |  |  |  |  |  |  |  |  |  |  |  |
| 0-1 | 792 /10598 | Reference |  |  | 177 /10598 | Reference |  |  |  |  |  |
| 2-3 | 6021 /109536 | 0.89 (0.82, 0.96) | 0.004 |  | 1206 /109536 | 0.85 (0.72, 0.99) | 0.049 |  |  |  |  |
| 4-7 | 11643 /216499 | 0.87 (0.81, 0.94) | < 0.001 |  | 2280 /216499 | 0.83 (0.70, 0.97) | 0.021 |  |  |  |  |
| >7 | 3145 /57649 | 0.89 (0.81, 0.96) | 0.004 |  | 632 /57649 | 0.83 (0.69, 0.99) | 0.040 |  |  |  |  |
| raw/total ratio |  |  |  |  |  |  |  |  |  |  |  |
| <0.33 | 6040 /91795 | Reference |  |  | 1228 /91795 | Reference |  |  |  |  |  |
| 0.33-0.42 | 5760 /107958 | 0.94 (0.91, 0.98) | 0.002 |  | 1132 /107958 | 0.97 (0.89, 1.05) | 0.454 |  |  |  |  |
| 0.43-0.50 | 5741 /110982 | 0.96 (0.93, 0.99) | 0.047 |  | 1168 /110982 | 1.02 (0.94, 1.11) | 0.572 |  |  |  |  |
| >0.50 | 4060 /83547 | 0.95 (0.91, 0.99) | 0.020 |  | 767 /83547 | 0.96 (0.87, 1.06) | 0.405 |  |  |  |  |

CVD: cardiovascular diseases. MI: myocardial infarction. HR: hazard ratio. CI: confidence interval. Vegetable intake was measured as the number of tablespoons consumed per day. Raw/total ratio was calculated as the amount of raw vegetable divided by the amount of total vegetable intake. The model was stratified by age (<50, 50-60, ≥60 years), sex, ethnicity, and region, and adjusted for educational attainment, Townsend deprivation index (continuous), hypertension, diabetes, physical activity level, smoking, alcohol consumption, BMI (continuous), use of mineral supplements, use of vitamin supplements, aspirin/ibuprofen, antihypertensive drugs, statins, insulin treatment, intake of fresh fruits, red meat, processed meat, oily fish and non-oily fish.

## Supplementary table 7: Associations between vegetable intake with incident CVD, incident MI, incident stroke, all-cause mortality, and CVD mortality, after excluding the events occurring within two years after recruitment.

|  | **Incident CVD** | | |  | **Incident MI** | | |  | **Incident stroke** | | |
| --- | --- | --- | --- | --- | --- | --- | --- | --- | --- | --- | --- |
|  | Cases/total | HR (95%CI) | p |  | Cases/total | HR (95%CI) | p |  | Cases/total | HR (95%CI) | p |
| Total vegetable |  |  |  |  |  |  |  |  |  |  |  |
| 0-1 | 858 /15600 | Reference |  |  | 578 /15661 | Reference |  |  | 297 /15849 | Reference |  |
| 2-3 | 4527 /108032 | 0.93 (0.86, 1.00) | 0.058 |  | 2867 /108378 | 0.91 (0.83, 1.00) | 0.043 |  | 1722 /109224 | 0.98 (0.86, 1.11) | 0.739 |
| 4-7 | 8661 /213645 | 0.91 (0.85, 0.98) | 0.013 |  | 5357 /214282 | 0.89 (0.81, 0.97) | 0.01 |  | 3415 /215922 | 0.97 (0.86, 1.10) | 0.624 |
| >7 | 2368 /56852 | 0.92 (0.84, 0.99) | 0.036 |  | 1482 /57049 | 0.89 (0.81, 0.99) | 0.031 |  | 950 /57464 | 1.01 (0.88, 1.15) | 0.931 |
| P for trend: |  |  | 0.059 |  |  |  | 0.060 |  |  |  | 0.853 |
|  |  |  |  |  |  |  |  |  |  |  |  |
| Raw vegetable |  |  |  |  |  |  |  |  |  |  |  |
| 0 | 2199 /38636 | Reference |  |  | 1435 /38814 | Reference |  |  | 797 /39275 | Reference |  |
| 1-2 | 9212 /226146 | 0.92 (0.87, 0.96) | 0.001 |  | 5742 /226831 | 0.92 (0.86, 0.97) | 0.005 |  | 3602 /228540 | 0.96 (0.88, 1.04) | 0.282 |
| 3-4 | 3441 /88993 | 0.90 (0.85, 0.95) | 0.001 |  | 2138 /89231 | 0.92 (0.85, 0.98) | 0.016 |  | 1365 /89925 | 0.92 (0.84, 1.01) | 0.092 |
| >4 | 1562 /40354 | 0.91 (0.85, 0.98) | 0.010 |  | 969 /40494 | 0.92 (0.84, 1.00) | 0.056 |  | 620 /40719 | 0.94 (0.84, 1.05) | 0.272 |
| P for trend: |  |  | 0.009 |  |  |  | 0.100 |  |  |  | 0.173 |
|  |  |  |  |  |  |  |  |  |  |  |  |
| Cooked vegetable |  |  |  |  |  |  |  |  |  |  |  |
| 0 | 594 /11896 | Reference |  |  | 396 /11941 | Reference |  |  | 206 /12059 | Reference |  |
| 1-2 | 7353 /186598 | 0.99 (0.91, 1.08) | 0.811 |  | 4607 /187121 | 0.98 (0.88, 1.09) | 0.730 |  | 2846 /188461 | 1.01 (0.88, 1.17) | 0.851 |
| 3-4 | 6588 /154281 | 0.99 (0.91, 1.09) | 0.883 |  | 4088 /154797 | 0.98 (0.88, 1.09) | 0.681 |  | 2571 /156048 | 1.02 (0.87, 1.18) | 0.840 |
| >4 | 1879 /41354 | 0.99 (0.90, 1.09) | 0.807 |  | 1193 /41511 | 0.97 (0.86, 1.09) | 0.583 |  | 761 /41891 | 1.09 (0.93, 1.28) | 0.295 |
| P for trend: |  |  | 0.972 |  |  |  | 0.593 |  |  |  | 0.212 |
|  |  |  |  |  |  |  |  |  |  |  |  |
|  | **All-cause mortality** | | |  | **CVD mortality** | | |  |  | | |
|  | Cases/total | HR (95%CI) | p |  | Cases/total | HR (95%CI) | p |  |  |  |  |
| Total vegetable |  |  |  |  |  |  |  |  |  |  |  |
| 0-1 | 1248 /15796 | Reference |  |  | 255 /15869 | Reference |  |  |  |  |  |
| 2-3 | 5701 /109216 | 0.82 (0.77, 0.87) | < 0.001 |  | 1118 /109448 | 0.88 (0.76, 1.01) | 0.065 |  |  |  |  |
| 4-7 | 11029 /215885 | 0.80 (0.76, 0.86) | < 0.001 |  | 2107 /216326 | 0.85 (0.74, 0.98) | 0.024 |  |  |  |  |
| >7 | 2984 /57488 | 0.81 (0.76, 0.87) | < 0.001 |  | 591 /57608 | 0.86 (0.74, 1.01) | 0.065 |  |  |  |  |
| P for trend: |  |  | < 0.001 |  |  |  | 0.124 |  |  |  |  |
|  |  |  |  |  |  |  |  |  |  |  |  |
| Raw vegetable |  |  |  |  |  |  |  |  |  |  |  |
| 0 | 3081 /39228 | Reference |  |  | 617 /39372 | Reference |  |  |  |  |  |
| 1-2 | 11483 /228509 | 0.87 (0.83, 0.91) | < 0.001 |  | 2206 /228983 | 0.90 (0.82, 0.99) | 0.031 |  |  |  |  |
| 3-4 | 4485 /89889 | 0.87 (0.83, 0.91) | < 0.001 |  | 874 /90071 | 0.94 (0.84, 1.05) | 0.256 |  |  |  |  |
| >4 | 1913 /40759 | 0.82 (0.77, 0.88) | < 0.001 |  | 374 /40825 | 0.86 (0.75, 0.99) | 0.039 |  |  |  |  |
| P for trend: |  |  | < 0.001 |  |  |  | 0.198 |  |  |  |  |
|  |  |  |  |  |  |  |  |  |  |  |  |
| Cooked vegetable |  |  |  |  |  |  |  |  |  |  |  |
| 0 | 972 /12028 | Reference |  |  | 182 /12081 | Reference |  |  |  |  |  |
| 1-2 | 9204 /188390 | 0.76 (0.70, 0.81) | < 0.001 |  | 1772 /188785 | 0.89 (0.76, 1.04) | 0.148 |  |  |  |  |
| 3-4 | 8327 /156059 | 0.77 (0.71, 0.82) | < 0.001 |  | 1594 /156383 | 0.87 (0.74, 1.03) | 0.110 |  |  |  |  |
| >4 | 2459 /41908 | 0.82 (0.75, 0.88) | < 0.001 |  | 523 /42002 | 0.98 (0.81, 1.17) | 0.786 |  |  |  |  |
| P for trend: |  |  | 0.541 |  |  |  | 0.636 |  |  |  |  |

CVD: cardiovascular diseases. MI: myocardial infarction. HR: hazard ratio. CI: confidence interval. Vegetable intake was measured as the number of tablespoons consumed per day. 1 unit of vegetable intake: one heaped tablespoon per day. The model was stratified by age (<50, 50-60, ≥60 years), sex, ethnicity, and region, and adjusted for educational attainment, Townsend deprivation index (continuous), hypertension, diabetes, physical activity level, smoking, alcohol consumption, BMI (continuous), use of mineral supplements, use of vitamin supplements, aspirin/ibuprofen, antihypertensive drugs, statins, insulin treatment, intake of fresh fruits, red meat, processed meat, oily fish and non-oily fish.

## Supplementary table 8: Associations between vegetable intake with incident CVD, incident MI, incident stroke, all-cause mortality, and CVD mortality in White population (n = 378 028).

|  | **Incident CVD** | | |  | **Incident MI** | | |  | **Incident stroke** | | |
| --- | --- | --- | --- | --- | --- | --- | --- | --- | --- | --- | --- |
|  | Cases/total | HR (95%CI) | p |  | Cases/total | HR (95%CI) | p |  | Cases/total | HR (95%CI) | p |
| Total vegetable |  |  |  |  |  |  |  |  |  |  |  |
| 0-1 | 916 /14605 | reference |  |  | 620 /14631 | reference |  |  | 313 /14759 | reference |  |
| 2-3 | 4785 /103709 | 0.91 (0.84, 0.98) | 0.009 |  | 3016 /103869 | 0.88 (0.80, 0.96) | 0.004 |  | 1823 /104581 | 0.97 (0.86, 1.09) | 0.601 |
| 4-7 | 9068 /204440 | 0.89 (0.83, 0.95) | 0.001 |  | 5623 /204757 | 0.86 (0.79, 0.94) | 0.001 |  | 3557 /206086 | 0.95 (0.84, 1.07) | 0.397 |
| >7 | 2353 /51628 | 0.90 (0.83, 0.97) | 0.008 |  | 1454 /51715 | 0.86 (0.78, 0.95) | 0.004 |  | 955 /52060 | 1.00 (0.87, 1.14) | 0.988 |
| P for trend: |  |  | 0.002 |  |  |  | 0.001 |  |  |  | 0.517 |
|  |  |  |  |  |  |  |  |  |  |  |  |
| Raw vegetable |  |  |  |  |  |  |  |  |  |  |  |
| 0 | 2370 /36669 | reference |  |  | 1543 /36742 | reference |  |  | 856 /37093 | reference |  |
| 1-2 | 9701 /216945 | 0.90 (0.86, 0.94) | < 0.001 |  | 6058 /217291 | 0.90 (0.85, 0.95) | < 0.001 |  | 3770 /218699 | 0.93 (0.86, 1.01) | 0.082 |
| 3-4 | 3529 /84103 | 0.87 (0.82, 0.92) | < 0.001 |  | 2184 /84203 | 0.88 (0.82, 0.95) | < 0.001 |  | 1404 /84764 | 0.89 (0.82, 0.98) | 0.016 |
| >4 | 1522 /36665 | 0.87 (0.82, 0.94) | < 0.001 |  | 928 /36736 | 0.87 (0.80, 0.95) | 0.002 |  | 618 /36930 | 0.91 (0.81, 1.02) | 0.098 |
| P for trend: |  |  | 0.001 |  |  |  | 0.012 |  |  |  | 0.117 |
|  |  |  |  |  |  |  |  |  |  |  |  |
| Cooked vegetable |  |  |  |  |  |  |  |  |  |  |  |
| 0 | 606 /10830 | reference |  |  | 399 /10849 | reference |  |  | 218 /10932 | reference |  |
| 1-2 | 7696 /178983 | 0.98 (0.90, 1.07) | 0.654 |  | 4825 /179251 | 0.98 (0.88, 1.09) | 0.719 |  | 2952 /180328 | 0.97 (0.84, 1.11) | 0.627 |
| 3-4 | 6929 /147582 | 1.00 (0.91, 1.09) | 0.933 |  | 4304 /147823 | 0.99 (0.89, 1.10) | 0.841 |  | 2701 /148865 | 0.99 (0.85, 1.14) | 0.838 |
| >4 | 1891 /36987 | 1.02 (0.93, 1.12) | 0.712 |  | 1185 /37049 | 1.00 (0.89, 1.12) | 0.964 |  | 777 /37361 | 1.09 (0.93, 1.27) | 0.287 |
| P for trend: |  |  | 0.182 |  |  |  | 0.660 |  |  |  | 0.012 |
|  |  |  |  |  |  |  |  |  |  |  |  |
|  | **All-cause mortality** | | |  | **CVD mortality** | | |  |  | | |
|  | Cases/total | HR (95%CI) | p |  | Cases/total | HR (95%CI) | p |  |  |  |  |
| Total vegetable |  |  |  |  |  |  |  |  |  |  |  |
| 0-1 | 1289 /14782 | reference |  |  | 275 /14782 | reference |  |  |  |  |  |
| 2-3 | 5851 /104731 | 0.81 (0.76, 0.86) | < 0.001 |  | 1161 /104731 | 0.83 (0.73, 0.95) | 0.009 |  |  |  |  |
| 4-7 | 11269 /206372 | 0.80 (0.75, 0.85) | < 0.001 |  | 2202 /206372 | 0.83 (0.72, 0.95) | 0.005 |  |  |  |  |
| >7 | 2930 /52143 | 0.80 (0.75, 0.86) | < 0.001 |  | 574 /52143 | 0.82 (0.70, 0.95) | 0.009 |  |  |  |  |
| P for trend: |  |  | < 0.001 |  |  |  | 0.004 |  |  |  |  |
|  |  |  |  |  |  |  |  |  |  |  |  |
| Raw vegetable |  |  |  |  |  |  |  |  |  |  |  |
| 0 | 3171 /37163 | reference |  |  | 653 /37163 | reference |  |  |  |  |  |
| 1-2 | 11775 /219012 | 0.87 (0.83, 0.90) | < 0.001 |  | 2306 /219012 | 0.89 (0.81, 0.98) | 0.018 |  |  |  |  |
| 3-4 | 4543 /84857 | 0.87 (0.83, 0.92) | < 0.001 |  | 893 /84857 | 0.93 (0.83, 1.03) | 0.177 |  |  |  |  |
| >4 | 1850 /36996 | 0.81 (0.76, 0.87) | < 0.001 |  | 360 /36996 | 0.84 (0.73, 0.97) | 0.016 |  |  |  |  |
| P for trend: |  |  | < 0.001 |  |  |  | 0.122 |  |  |  |  |
|  |  |  |  |  |  |  |  |  |  |  |  |
| Cooked vegetable |  |  |  |  |  |  |  |  |  |  |  |
| 0 | 981 /10951 | reference |  |  | 185 /10951 | reference |  |  |  |  |  |
| 1-2 | 9459 /180574 | 0.75 (0.70, 0.80) | < 0.001 |  | 1853 /180574 | 0.89 (0.76, 1.05) | 0.160 |  |  |  |  |
| 3-4 | 8494 /149084 | 0.76 (0.70, 0.81) | < 0.001 |  | 1662 /149084 | 0.88 (0.75, 1.04) | 0.131 |  |  |  |  |
| >4 | 2405 /37419 | 0.81 (0.75, 0.88) | < 0.001 |  | 512 /37419 | 0.98 (0.82, 1.17) | 0.786 |  |  |  |  |
| P for trend: |  |  | 0.891 |  |  |  | 0.340 |  |  |  |  |

CVD: cardiovascular diseases. MI: myocardial infarction. HR: hazard ratio. CI: confidence interval. Vegetable intake was measured as the number of tablespoons consumed per day. 1 unit of vegetable intake: one heaped tablespoon per day. The model was stratified by age (<50, 50-60, ≥60 years), sex, and region, and adjusted for educational attainment, Townsend deprivation index (continuous), hypertension, diabetes, physical activity level, smoking, alcohol consumption, BMI (continuous), use of mineral supplements, use of vitamin supplements, aspirin/ibuprofen, antihypertensive drugs, statins, insulin treatment, intake of fresh fruits, red meat, processed meat, oily fish and non-oily fish.

## Supplementary table 9: Associations between vegetable intake with incident CVD, incident MI, incident stroke, all-cause mortality, and CVD mortality in non-White population (n = 21 558).

|  | **Incident CVD** | | |  | **Incident MI** | | |  | **Incident stroke** | | |
| --- | --- | --- | --- | --- | --- | --- | --- | --- | --- | --- | --- |
|  | Cases/total | HR (95%CI) | p |  | Cases/total | HR (95%CI) | p |  | Cases/total | HR (95%CI) | p |
| Total vegetable |  |  |  |  |  |  |  |  |  |  |  |
| 0-1 | 57 /1106 | reference |  |  | 41 /1109 | reference |  |  | 15 /1117 | reference |  |
| 2-3 | 188 /4754 | 0.93 (0.69, 1.26) | 0.651 |  | 125 /4767 | 0.92 (0.64, 1.31) | 0.632 |  | 65 /4793 | 1.12 (0.63, 1.98) | 0.706 |
| 4-7 | 439 /10038 | 1.05 (0.78, 1.40) | 0.753 |  | 275 /10052 | 1.00 (0.71, 1.42) | 0.982 |  | 152 /10115 | 1.19 (0.68, 2.06) | 0.542 |
| >7 | 246 /5450 | 1.02 (0.76, 1.39) | 0.885 |  | 163 /5464 | 1.04 (0.72, 1.50) | 0.827 |  | 89 /5493 | 1.21 (0.68, 2.14) | 0.513 |
| P for trend: | 930 /21348 | 1.00 (0.97, 1.03) | 0.970 |  |  |  | 0.925 |  |  |  | 0.564 |
|  |  |  |  |  |  |  |  |  |  |  |  |
| Raw vegetable |  |  |  |  |  |  |  |  |  |  |  |
| 0 | 110 /2240 | reference |  |  | 74 /2246 | reference |  |  | 36 /2268 | reference |  |
| 1-2 | 395 /10065 | 0.95 (0.76, 1.19) | 0.657 |  | 251 /10085 | 0.99 (0.75, 1.30) | 0.917 |  | 142 /10130 | 0.92 (0.63, 1.35) | 0.677 |
| 3-4 | 252 /5223 | 1.21 (0.95, 1.54) | 0.124 |  | 166 /5233 | 1.39 (1.03, 1.87) | 0.030 |  | 80 /5272 | 0.94 (0.62, 1.43) | 0.775 |
| >4 | 173 /3820 | 1.08 (0.83, 1.41) | 0.556 |  | 113 /3828 | 1.16 (0.84, 1.61) | 0.362 |  | 63 /3848 | 1.05 (0.67, 1.65) | 0.818 |
| P for trend: |  |  | 0.140 |  |  |  | 0.076 |  |  |  | 0.600 |
|  |  |  |  |  |  |  |  |  |  |  |  |
| Cooked vegetable |  |  |  |  |  |  |  |  |  |  |  |
| 0 | 65 /1138 | reference |  |  | 50 /1140 | reference |  |  | 15 /1149 | reference |  |
| 1-2 | 334 /8274 | 0.86 (0.65, 1.14) | 0.301 |  | 224 /8292 | 0.79 (0.57, 1.09) | 0.150 |  | 115 /8336 | 1.16 (0.66, 2.04) | 0.598 |
| 3-4 | 332 /7363 | 0.90 (0.68, 1.20) | 0.464 |  | 193 /7374 | 0.72 (0.51, 1.00) | 0.052 |  | 125 /7426 | 1.32 (0.75, 2.33) | 0.334 |
| >4 | 199 /4573 | 0.80 (0.59, 1.08) | 0.152 |  | 137 /4586 | 0.74 (0.52, 1.06) | 0.098 |  | 66 /4607 | 1.03 (0.57, 1.87) | 0.916 |
| P for trend: |  |  | 0.252 |  |  |  | 0.251 |  |  |  | 0.665 |
|  |  |  |  |  |  |  |  |  |  |  |  |
|  | **All-cause mortality** | | |  | **CVD mortality** | | |  |  | | |
|  | Cases/total | HR (95%CI) | p |  | Cases/total | HR (95%CI) | p |  |  |  |  |
| Total vegetable |  |  |  |  |  |  |  |  |  |  |  |
| 0-1 | 65 /1120 | reference |  |  | 13 /1120 | reference |  |  |  |  |  |
| 2-3 | 170 /4805 | 0.73 (0.54, 0.98) | 0.035 |  | 45 /4805 | 1.04 (0.55, 1.96) | 0.907 |  |  |  |  |
| 4-7 | 374 /10127 | 0.71 (0.54, 0.94) | 0.016 |  | 78 /10127 | 0.85 (0.46, 1.57) | 0.596 |  |  |  |  |
| >7 | 215 /5506 | 0.71 (0.52, 0.95) | 0.022 |  | 58 /5506 | 1.14 (0.60, 2.16) | 0.686 |  |  |  |  |
| P for trend: |  |  | 0.014 |  |  |  | 0.927 |  |  |  |  |
|  |  |  |  |  |  |  |  |  |  |  |  |
| Raw vegetable |  |  |  |  |  |  |  |  |  |  |  |
| 0 | 118 /2273 | reference |  |  | 28 /2273 | reference |  |  |  |  |  |
| 1-2 | 360 /10149 | 0.84 (0.67, 1.04) | 0.114 |  | 78 /10149 | 0.82 (0.52, 1.31) | 0.411 |  |  |  |  |
| 3-4 | 190 /5280 | 0.79 (0.62, 1.02) | 0.072 |  | 47 /5280 | 0.93 (0.56, 1.56) | 0.789 |  |  |  |  |
| >4 | 156 /3856 | 0.86 (0.66, 1.12) | 0.266 |  | 41 /3856 | 1.00 (0.58, 1.74) | 0.989 |  |  |  |  |
| P for trend: |  |  | 0.682 |  |  |  | 0.460 |  |  |  |  |
|  |  |  |  |  |  |  |  |  |  |  |  |
| Cooked vegetable |  |  |  |  |  |  |  |  |  |  |  |
| 0 | 65 /1151 | reference |  |  | 18 /1151 | reference |  |  |  |  |  |
| 1-2 | 282 /8353 | 0.70 (0.53, 0.93) | 0.015 |  | 61 /8353 | 0.54 (0.31, 0.95) | 0.032 |  |  |  |  |
| 3-4 | 293 /7435 | 0.78 (0.58, 1.04) | 0.095 |  | 68 /7435 | 0.66 (0.37, 1.18) | 0.160 |  |  |  |  |
| >4 | 184 /4619 | 0.74 (0.54, 1.01) | 0.056 |  | 47 /4619 | 0.68 (0.37, 1.24) | 0.208 |  |  |  |  |
| P for trend: |  |  | 0.658 |  |  |  | 0.894 |  |  |  |  |

CVD: cardiovascular diseases. MI: myocardial infarction. HR: hazard ratio. CI: confidence interval. Vegetable intake was measured as the number of tablespoons consumed per day. 1 unit of vegetable intake: one heaped tablespoon per day. The model was stratified by age (<50, 50-60, ≥60 years), sex, and region, and adjusted for educational attainment, Townsend deprivation index (continuous), hypertension, diabetes, physical activity level, smoking, alcohol consumption, BMI (continuous), use of mineral supplements, use of vitamin supplements, aspirin/ibuprofen, antihypertensive drugs, statins, insulin treatment, intake of fresh fruits, red meat, processed meat, oily fish and non-oily fish.

## Supplementary table 10: The basic characteristics and main findings of the previous studies investigating the independent effects of raw vs. cooked vegetable intake.

| Study | Sample size | Follow-up (yrs) | Age (yrs) | Male % | Exposure assessment | Covariate adjustment | Outcome  (event number) | Findings |
| --- | --- | --- | --- | --- | --- | --- | --- | --- |
| PURE ^1^ | 135 335 | 7.4 | 50.0 | 42 | Country-specific FFQ in 18 countries. Cooked vegetables did not include potatoes or legumes. | Age, gender, energy intake, smoking, residence location, household income, physical activity, diabetes, education, dietary factors (meat, bread, cereal) | 1. All cause death (5 796) | Raw vegetable intake reduced death risk, but cooked vegetable did not. |
|  |  |  |  |  |  |  | 2. Major CVD events (4 784) | Neither reduced major CVD incidence. |
|  |  |  |  |  |  |  |  |  |
| EPIC ^2^ | 451 151 | 13 | 51.2 | 29 | Country-specific FFQ in 10 countries. Cooked vegetables did not include potatoes or legumes. | Physical activity, education, smoking, processed meat intake | 1. All cause death (25 682) | Both reduced all cause death, higher reduction with raw vegetable intake than cooked vegetable. |
|  |  |  |  |  |  |  | 2. CVD mortality (5 125) | Both reduced CVD mortality, higher reduction with raw vegetable intake than cooked vegetable. |
|  |  |  |  |  |  |  |  |  |
| Australian cohort ^3^ | 150 969 | 6.2 | 60.0 | 45 | A short dietary questionnaire. Cooked vegetables included potato. | Age, gender, education, marital status, residence, socio-economic status, smoking, physical activity, body mass index, dietary factors (multi-vitamin use, processed meat intake, diabetes, fruit intake) | 1. All cause death (6 038) | Cooked vegetable intake reduced all cause death, but  raw vegetable did not. |
|  |  |  |  |  |  |  |  |  |
| MORGEN (Dutch) cohort ^4^ | 20 069 | 10.3 | 41.5 | 55 | Dutch FFQ (as part of EPIC study). Cooked vegetables did not include potatoes or legumes (except French beans). | Age, gender, total energy intake, smoking, alcohol, education, hormone replacement therapy, family history of CVDs, body mass index, dietary factors (whole grain foods, processed meat, fish, fruit, supplement use) | 1. Stroke (233) | Raw vegetable intake reduced stroke risk but cooked vegetable did not. |

1. Miller V, Mente A, Dehghan M, et al. Fruit, vegetable, and legume intake, and cardiovascular disease and deaths in 18 countries (PURE): a prospective cohort study. Lancet 2017;390:2037–49. doi:10.1016/S0140-6736(17)32253-5

2. Leenders M, Sluijs I, Ros MM, et al. Fruit and vegetable consumption and mortality: European prospective investigation into cancer and nutrition. Am J Epidemiol 2013;178:590–602. doi:10.1093/aje/kwt006

3. Nguyen B, Bauman A, Gale J, et al. Fruit and vegetable consumption and all-cause mortality: evidence from a large Australian cohort study. Int J Behav Nutr Phys Act 2016;13:9. doi:10.1186/s12966-016-0334-5

4. Oude Griep LM, Verschuren WMM, Kromhout D, et al. Raw and processed fruit and vegetable consumption and 10-year stroke incidence in a population-based cohort study in the Netherlands. Eur J Clin Nutr 2011;65:791–9. doi:10.1038/ejcn.2011.36

## Supplementary figure 1: the distributions of intakes of raw, cooked and total vegetable in UK Biobank cohort. (number of heaped tablespoons/day)

| 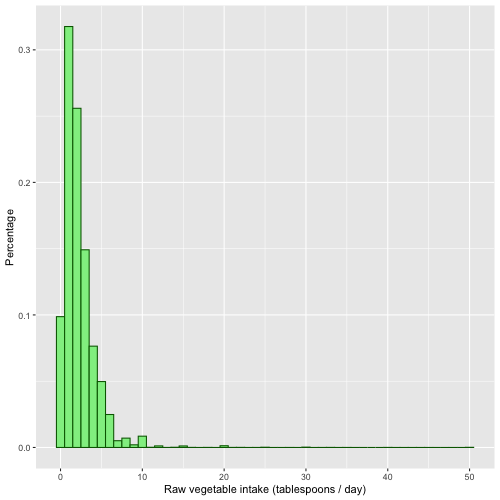 | 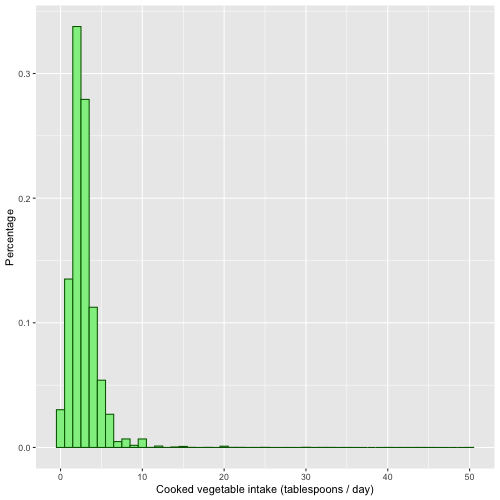 |
| --- | --- |
| 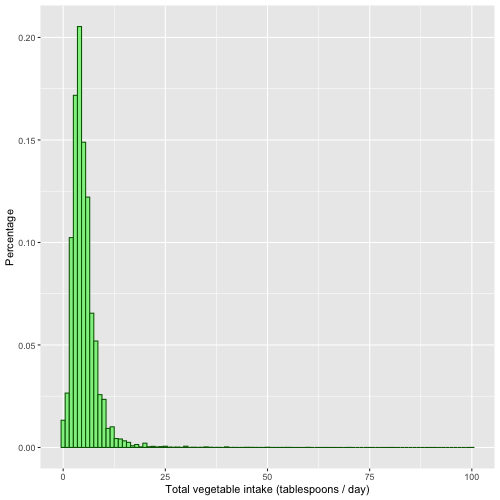 | \|  \| Raw vegetable intake \| Cooked vegetable intake \| Total vegetable intake \| \| --- \| --- \| --- \| --- \| \| Min \| 0 \| 0 \| 0 \| \| 10% \| 1 \| 1 \| 2 \| \| 20% \| 1 \| 2 \| 3 \| \| 30% \| 1 \| 2 \| 3 \| \| 40% \| 1 \| 2 \| 4 \| \| Median \| 2 \| 2 \| 4 \| \| Mean \| 2.27 \| 2.78 \| 5.05 \| \| 60% \| 2 \| 3 \| 5 \| \| 70% \| 3 \| 3 \| 6 \| \| 80% \| 3 \| 4 \| 7 \| \| 90% \| 5 \| 5 \| 8 \| \| Max \| 50 \| 50 \| 100 \| |

Upper-left: the histogram for raw vegetable intake distribution.

Upper-right: the histogram for cooked vegetable intake distribution.

Lower-left: the histogram for total vegetable intake.

Lower-right: the table showing the distributions of vegetable intakes


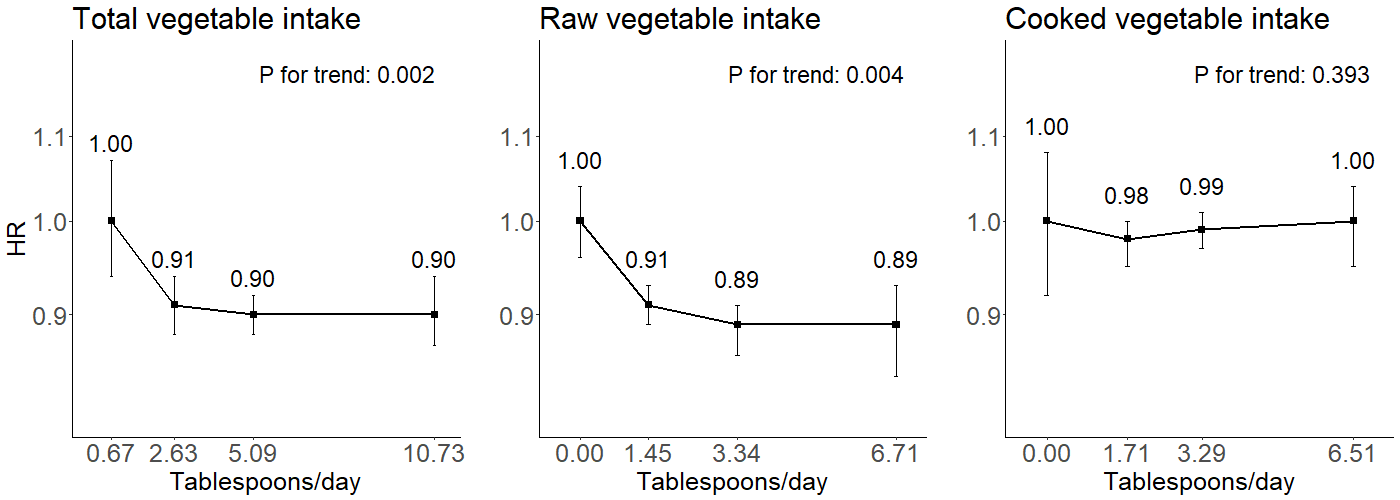


## Supplementary figure 2: Association of incident CVD with total, raw and cooked vegetable consumption

Hazard ratios (HR; fully adjusted models) and their 95% float absolute risk estimated confidence interval by level of vegetable consumption (heaped tablespoons/day). Exclusions as in Table 1. The numbers above confidence bars are HR estimates. The numbers under the X axis are the mean number of heaped tablespoons/day in each level of vegetable intake. CVD: cardiovascular disease. Floating absolute risk estimates provide variance for each exposure level (including the reference level) to facilitate comparison across all exposure levels (see Plummer M. Stat Med 2004;23(1):93-104).
